# Multi-omic profiling of pituitary thyrotropic cells and progenitors

**DOI:** 10.1186/s12915-021-01009-0

**Published:** 2021-04-15

**Authors:** Alexandre Z. Daly, Lindsey A. Dudley, Michael T. Peel, Stephen A. Liebhaber, Stephen C. J. Parker, Sally A. Camper

**Affiliations:** 1 Department Human Genetics, University of Michigan Medical School, Ann Arbor, MI 48109 USA; 2 Department Genetics, University of Pennsylvania Perelman School of Medicine, Ann Arbor, MI 48109 USA; 3 Incyte, Wilmington, DE 19803 USA; 4 Department Computational Medicine and Bioinformatics, University of Michigan Medical School, Ann Arbor, MI 48109 USA

**Keywords:** TSH, Chromatin, bZIP, bHLH, bHTH, POU1F1, GATA2

## Abstract

**Background:**

The pituitary gland is a neuroendocrine organ containing diverse cell types specialized in secreting hormones that regulate physiology. Pituitary thyrotropes produce thyroid-stimulating hormone (TSH), a critical factor for growth and maintenance of metabolism. The transcription factors POU1F1 and GATA2 have been implicated in thyrotrope fate, but the transcriptomic and epigenomic landscapes of these neuroendocrine cells have not been characterized. The goal of this work was to discover transcriptional regulatory elements that drive thyrotrope fate.

**Results:**

We identified the transcription factors and epigenomic changes in chromatin that are associated with differentiation of POU1F1-expressing progenitors into thyrotropes using cell lines that represent an undifferentiated *Pou1f1* lineage progenitor (GHF-T1) and a committed thyrotrope line that produces TSH (TαT1). We compared RNA-seq, ATAC-seq, histone modification (H3K27Ac, H3K4Me1, and H3K27Me3), and POU1F1 binding in these cell lines. POU1F1 binding sites are commonly associated with bZIP transcription factor consensus binding sites in GHF-T1 cells and Helix-Turn-Helix (HTH) or basic Helix-Loop-Helix (bHLH) factors in TαT1 cells, suggesting that these classes of transcription factors may recruit or cooperate with POU1F1 binding at unique sites. We validated enhancer function of novel elements we mapped near *Cga, Pitx1, Gata2,* and *Tshb* by transfection in TαT1 cells. Finally, we confirmed that an enhancer element near *Tshb* can drive expression in thyrotropes of transgenic mice, and we demonstrate that GATA2 enhances *Tshb* expression through this element.

**Conclusion:**

These results extend the ENCODE multi-omic profiling approach to the pituitary gland, which should be valuable for understanding pituitary development and disease pathogenesis.

**Graphical abstract:**

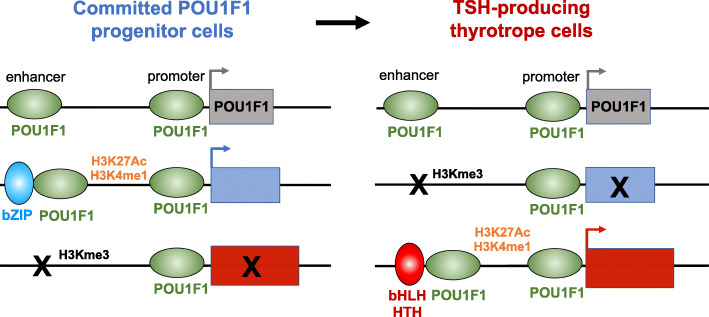

## Background

Recent genome-wide association studies (GWAS) have begun to identify loci that are associated with sporadic pituitary adenomas and variation in normal height, but the genes associated with many of these loci are unknown [1–3]. Nearly 90% of GWAS hits are in noncoding regions, making it difficult to transition from genetic mapping to biological mechanism [4]. Recent studies that identify enhancer regions by undertaking large-scale functional genomic annotation of noncoding elements like Encyclopedia of DNA Elements (ENCODE) have begun to yield a better understanding of some complex phenotypes and diseases. Dense molecular profiling maps of the transcriptome and epigenome have been generated for more than 250 cell lines and 150 tissues, but pituitary cell lines or tissues were not included. This represents a major limitation, as the cell types that comprise the pituitary gland secrete hormones responsible for growth (growth hormone secreted by somatotropes), reproduction (gonadotropins secreted by gonadotropes), adrenal gland function and the stress response (ACTH secreted by corticotropes), lactation (prolactin secreted by lactotropes), and thyroid gland function (thyroid-stimulating hormone secreted by thyrotropes). Epigenomic and gene expression data are emerging for somatotropes, gonadotropes, and corticotropes, but there is very little available data on thyrotropes [5–8].

Thyrotropes represent ~ 5% of cells in the pituitary gland, and their function is to express and secrete thyroid-stimulating hormone (TSH or thyrotropin), which regulates thyroid gland development and thyroid hormone production. These hormones are essential for normal growth and metabolism. Up to 12% of the US population has abnormal levels of thyrotropin [9]. The incidence of secondary hypothyroidism is estimated to be 1:20,000 to 1:80,000 individuals [10]. Research into the regulation of thyrotrope differentiation and function is relevant to this public health problem.

A cascade of transcription factors is responsible for the differentiation of the major pituitary hormone-producing cell types. The transcription factors associated with thyrotrope development and function are POU1F1, GATA2, ISL1, PITX1, and PITX2*.* The pituitary transcription factor POU1F1 is essential for the differentiation of growth hormone, prolactin, and TSH-producing cells [11]. It binds to the promoters of *Gh, Prl,* and *Tshb* to activate gene expression [12–16]. Defects in the POU1F1 gene cause severe growth insufficiency and hypothyroidism in humans and mice [11, 17]. POU1F1 and GATA2 act synergistically to activate *Tshb* expression through promoter-proximal elements [13, 18]. Defects in GATA2 and ISL1 reduce thyrotrope differentiation in mice, but they do not appear to ablate it [19–21]. Finally, induction of *Pitx2* deficiency in thyrotropes using *Tshb-cre* causes moderate growth deficiency, blunted TSH response to hypothyroidism challenge, and elevated *Pitx1* expression [22]. This suggests that *Pitx2* has a role in thyrotrope function that overlaps with the related *Pitx1* gene [23, 24]. Despite the important role of *Pou1f1* in thyrotrope development and function, little is known about the gene regulatory network of POU1F1 in progenitors or thyrotropes.

Due to the scarcity of the thyrotrope cell type, classical genomic techniques are challenging to apply. Hormone-producing cell lines have been invaluable for understanding changes in chromatin and gene expression that occur during development [5, 25]. To discover thyrotrope-specific regulatory elements and potential drivers of differentiation, we generated and compared the transcriptome (RNA-seq), open chromatin (ATAC-seq), histone modification (CUT&RUN for H3K27Ac, H3K4Me1, and H3K27Me3), and transcription factor binding (CUT&RUN for POU1F1) in two mouse cell lines, a POU1F1-expressing pituitary precursor cell line that does not express any hormones, GHF-T1, to a thyrotrope-like cell line, TαT1 [26, 27]. TαT1 cells behave much like endogenous thyrotropes in that they respond to TRH and retinoids, and they secrete TSH in response to diurnal cues [28–30]. Finally, we evaluated putative enhancer elements for function using transfection assays in TαT1 cells and genetically engineered mice. Together, these studies extend ENCODE-like multi-omic analyses to generate reference maps of gene regulation for cell types critical for growth and metabolism.

## Results

### Comparison of transcriptomes

**Fig. 1:**
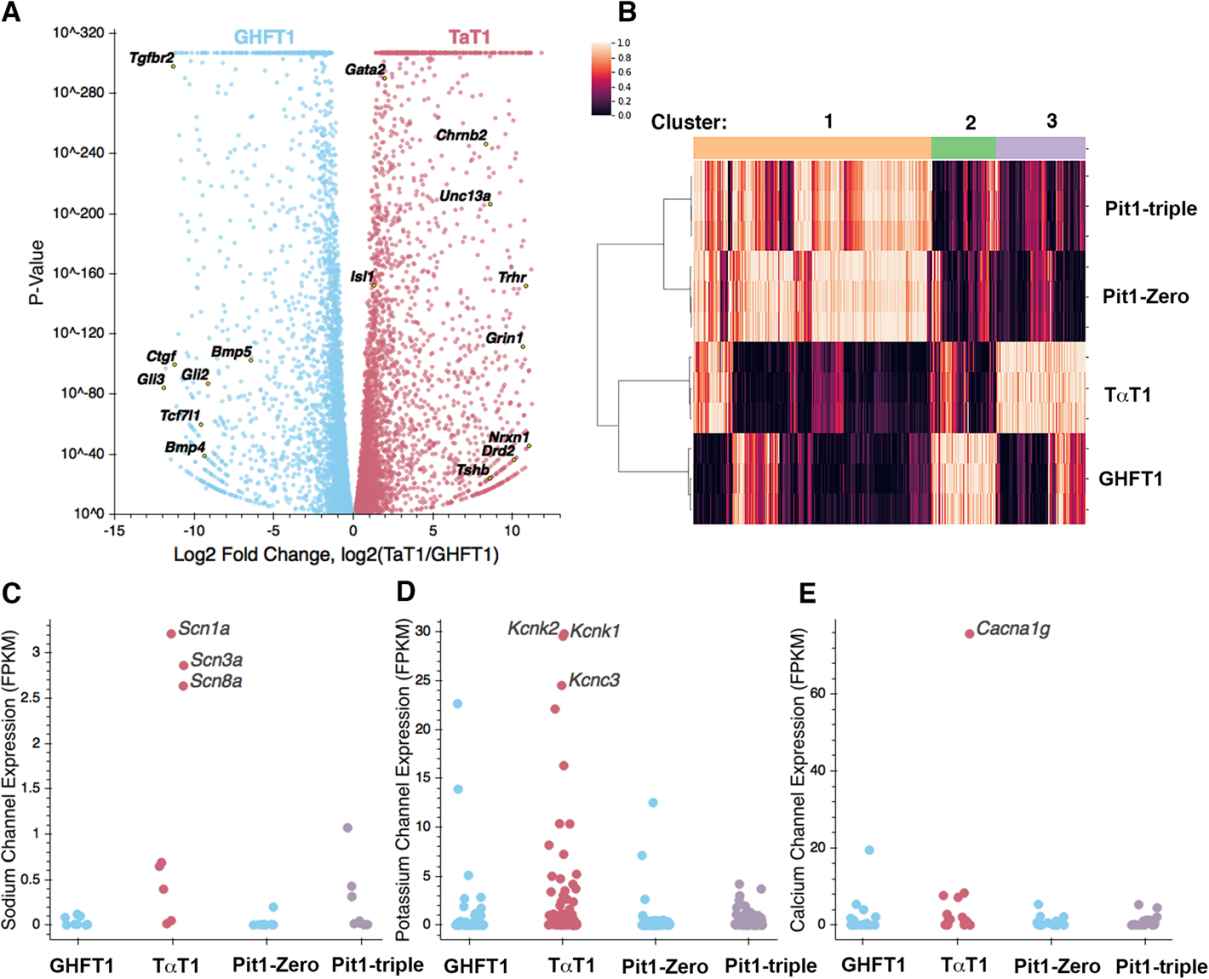
**a** Volcano plot of differential gene expression for GHF-T1 compared to TαT1 cells. Genes with elevated expression in GHF-T1 cells are colored blue, and those elevated in TαT1 cells are colored red. Labeled genes represent key factors and genes associated with GO terms in Table 2. **b** Heatmap showing similarly and differentially expressed genes across GHF-T1, TαT1, Pit1-Zero, and Pit1-Triple cells. Genes associated with each cluster are presented in Supplemental Table 1. **c** FPKM values of neuronal sodium channel genes. **d** FPKM values of potassium channel genes. **e** FPKM values of calcium channel genes. *N* = 3/cell line

To identify candidate factors that drive the differentiation of thyrotropes, we performed RNA sequencing on the GHF-T1 and TαT1 cell lines. There were many differences in their transcriptomes, consistent with their distinctive morphology, growth rate, and hormone secretion properties (Fig. 1a). Eighty-two percent of genes were differentially expressed (FDR < 0.01). *Pou1f1* expression levels were nearly twice as high in TαT1 cells (160 FPKM) relative to GHF-T1 cells (85 FPKM). Other SV40 immortalized pituitary cell lines vary ten-fold in *Pou1f1* expression levels, but there was no correlation with differentiation state [31]. As expected, *Cga* and *Tshb* were expressed in TαT1 (3557 and 11 FPKM, respectively) but negligibly expressed in GHF-T1 cells (1.4 and 0 FPKM, respectively). The GHF-T1 cells had elevated expression of the transcription factors *Gli3*, *Pax3*, *Foxg1,* and others (Table 1). TαT1 cells had elevated expression of *Gata2* and *Isl1,* as expected, and *Lhx3*, *Rxrg*, *Neurod4, Insm1,* and other transcription factors were also expressed at significantly higher levels than in GHFT1 cells. Some of the differences in transcription factor gene expression were dramatic, i.e., in the range of 8–10 fold, but others were more modest, like *Isl1* (1.3x). *Isl1* is critical for driving thyrotrope fate, but it is expressed in both progenitors and differentiated cells [19].

**Table 1:** Differentially expressed transcription factors (FDR < 5 × 10^− 14^)

GHFT1 cells			TαT1 cells		
Gene	Log2 fold-change	Rank	Gene	Log2 fold-change	Rank
*Gli3*	11.90	1	*Lhx3*	11.04	8
*Pax3*	10.95	12	*Rxrg*	10.74	25
*Zfhx4*	10.74	21	*Insm1*	10.67	30
*Foxg1*	10.59	26	*Neurod4*	10.56	37
*Zfp57*	10.51	29	*Sox3*	10.23	55
*Hoxc13*	10.29	40	*Fev*	10.18	57
*Kdm5D*	10.19	48	*Myt1l*	9.93	72
*Hmga2*	10.15	53	*Scrt2*	9.90	77
*Msx2*	10.11	54	*Zfp641*	9.39	126
*Runx1*	10.07	55	*Scrt1*	9.15	145
*Rhox10*	9.94	60	*Zic3*	8.96	168
*Mecom*	9.84	68	*En2*	8.95	169
*Tead2*	9.65	80	*Lin28b*	8.82	184
*Vax1*	9.58	85	*Pax5*	8.61	205
*Tcf7l1*	9.57	87	*Zfp709*	8.52	218
*Hoxa1*	9.52	91	*Prdm16*	8.30	249
*Tcf24*	9.46	95	*Pou2f2*	8.15	268
*Hoxc9*	9.35	104	*Zim1*	7.92	301
*Maf*	9.32	106	*Nhlh1*	7.85	312
*Pou3f3*	9.16	112	*Foxl2*	7.82	317
*Gli2*	9.13	117	*Isl1*	1.3	3896

To uncover pathways with altered gene expression in these cell lines, we performed GO term (gene ontology) enrichment analysis on the top 5% of the most differentially expressed genes (by log-2 fold-change) in both lines [32, 33]. The GO terms enriched in GHFT1 cells were broadly related to development and morphogenesis (Table 2). Genes contributing to these GO terms include genes from the GLI family that are targets of hedgehog signaling (*Gli2*, *Gli3*, *Glipr1*, *Glipr2*, *Glis2*, *Glis3*), BMPs (*Bmp1*, *Bmp4*, *Bmp5*, *Bmpr1a*, *Bmpr2*, *Bmper*, *Bmp2k*), and FGFs (*Fgf5*, *Fgf7*, *Fgf8*, *Fgf10*, and *Fgf21*). Elevated expression of these factors in GHFT1 cells is consistent with the underlying the importance of sonic hedgehog, BMP, and FGF signaling in early pituitary development [34–36]. In contrast, the genes with elevated expression in TαT1 cells were enriched for GO terms related to nervous system development and synapse formation. This may be attributable to the fact that the hormone secretory cells in the pituitary gland are excitable and fire action potentials, which are altered in response to hypothalamic input and exhibit characteristic patterns of hormone release [37]. In addition, stimulus-secretion coupling also involves interconnection of homotypic networks [38]. Some genes enriched in TαT1 cells that contribute to these neuronal-like GO terms are neurexin 1 (*Nrxn1*), genes of the glutamate receptor family (*Grin1*, *Grina*, *Grin2d*, *Grin3a*), and the synaptic regulator *Unc13a*. KEGG pathway enrichment analysis revealed an increase in neuroactive ligand-receptor interaction in TαT1 cells, consistent with the enrichment in GO terms found.

**Table 2:** Gene ontology term enrichment

GHFT1 cells								TαT1 cells							
Gene	Structure and development^**1**^					Expression		Gene	Synapse development and function^**2**^					Expression	
	A	B	C	D	E	GHFT1	TαT1		F	G	H	I	J	GHFT1	TαT1
*Cyr61*	X	X	X	X	X	117.1	0.5	*Nrxn1*	X	X	X	X	X	0.0	4.2
*Edn1*	X	X	X	X	X	7.3	0.0	*Unc13a*	X	X	X	X	X	0.0	19.8
*Bmp4*	X	X	X	X	X	6.8	0.0	*Drd2*	X	X		X	X	0.0	12.4
*Tgfbr2*	X	X	X	X	X	65.1	0.0	*Chrnb2*	X	X		X	X	0.0	12.7
*Fgf10*	X	X	X	X	X	1.1	0.0	*Grin1*	X	X		X	X	0.0	20.5
*Gli3*	X	X	X	X	X	13.4	0.0	*Syt4*	X	X		X	X	0.0	86.3
*Mef2c*	X	X	X	X	X	7.6	0.0	*Grin3a*	X	X		X	X	0.0	11.4
*Cav1*	X	X	X	X	X	10.1	0.0	*Snap25*	X	X		X	X	0.1	107.4
*Bmp5*	X	X	X	X	X	3.8	0.0	*Sez6*	X	X	X	X	X	0.0	39.8
*Ctgf*	X	X	X	X	X	40.3	0.0	*Gabrg2*	X	X	X	X	X	0.0	13.0
*Gli2*	X	X	X	X	X	4.2	0.0	*Pclo*	X	X	X	X	X	0.0	4.0
*Gata6*	X	X	X	X	X	1.2	0.0	*Atp2b2*			X	X		0.0	9.4
*Tbx20*	X	X	X	X	X	2.2	0.0	*Chrna4*	X	X			X	0.0	3.4
*Msx2*	X	X	X	X	X	20.1	0.0	*Shisa6*	X	X			X	0.0	11.3
*Yap1*	X	X	X	X	X	40.3	0.2	*Lrrc4c*			X	X		0.0	1.7
*Sfrp1*	X	X	X	X	X	7.1	0.0	*Thy1*				X		0.0	23.3
*Hlx*	X	X	X	X	X	1.5	0.0	*Glra3*	X	X	X		X	0.0	28.1
*Cxcl12*	X	X	X	X	X	4.2	0.0	*Adgfb1*			X	X		0.0	9.5
*Pax3*	X	X	X	X	X	9.1	0.0	*Gria1*	X	X			X	0.0	11.7
*Fn1*	X	X	X	X	X	611.8	1.0	*Lgi1*				X		0.0	1.1

^1^ The top five GO terms associated with the top 5% of the most differentially expressed genes (log 2-fold-change) in GHFT1 cells are A = animal organ development, B = anatomical structure development, C = anatomical structure morphogenesis, D = multicellular organism development, E = system development. X’s illustrate the association of each gene with a GO term. Expression is given in FPKM

^2^ The top five GO terms associated with TαT1 cells are: F = synaptic signaling, G = trans-synaptic signaling, H = synapse organization, I = nervous system development, J = anterograde trans-synaptic signaling

The function of several members of the bHLH family of transcription factors, including *Ascl1, Neurod4,* and *Neurod1*, has been investigated in pituitary development [39]. Seventy-one of the ninety-three known bHLH factors are differentially expressed between GHF-T1 and TαT1 cells (FDR < 0.05); *Neurod4* and *Ascl1* are expressed at higher levels in TαT1 cells (Supplemental Table 2). *Ascl1* is essential for development of all hormone-producing cell types in fish pituitary, and in mice, *Ascl1* loss of function causes reduced production of *Pomc*, *Lhb*, and *Fshb* [39, 40]. However, these reports conflict on whether thyrotropes are affected by *Ascl1* deficiency. We performed TSH immunostaining on pituitaries from *Ascl1*-null mice and did not detect a reduction in thyrotropes at e18.5 (Supplemental Figure 1), suggesting *Ascl1* is not required for thyrotrope cell specification. Repressive bHLH genes of the ID family had the highest expression in both of the cell lines, and the role of these genes has not been investigated.

We compared gene expression profiles that we obtained from GHF-T1 and TαT1 cells with those of other SV40-transformed pituitary cell lines, Pit1-zero and Pit1-triple cells (Fig. 1b) [31]. Pit1-zero and Pit1-triple cells were transformed using the same *Pou1f1* regulatory elements as the GHF-T1 cell line. Pit1-zero cells express *Pou1f1*, but none of POU1F1’s downstream hormone genes, whereas Pit1-triple cells express *Pou1f1* and all three POU1F1-dependent hormones, GH, PRL, and TSH. In the TαT1 cell line, we found a statistically significant increase in the expression of sodium channel genes (*p* value = 0.002) and potassium channel genes (*p* value = 2.9e−05), but not in calcium channel genes as a group (*p* value = 0.26) (Fig. 1c–e). The most highly expressed sodium channel genes in TαT1 cells are *Scn1a*, *Scn8a*, and *Scn3a*. Three sodium channel genes (*Scn1a*, *Scn8a*, and *Scn9a*) are also expressed in the Pit1-Triple cell line, the only other hormone-expressing cell lineage we studied. The most highly expressed potassium channel genes in TαT1 cells are *Kcnc3*, *Kcnq2*, *Kcnk1*, and *Kcnk2*. G protein-gated ion channels are involved in regulated hormone secretion. This marked increase in ion channel genes in the TαT1 cells is consistent with their GO terms associated with synapses and neuron formation and function. The only calcium channel gene with differential expression was *Cacna1g*, which is highly expressed in TαT1 cells relative to the other three cell lines.

### Chromatin landscape around thyrotrope-signature genes

**Fig. 2:**
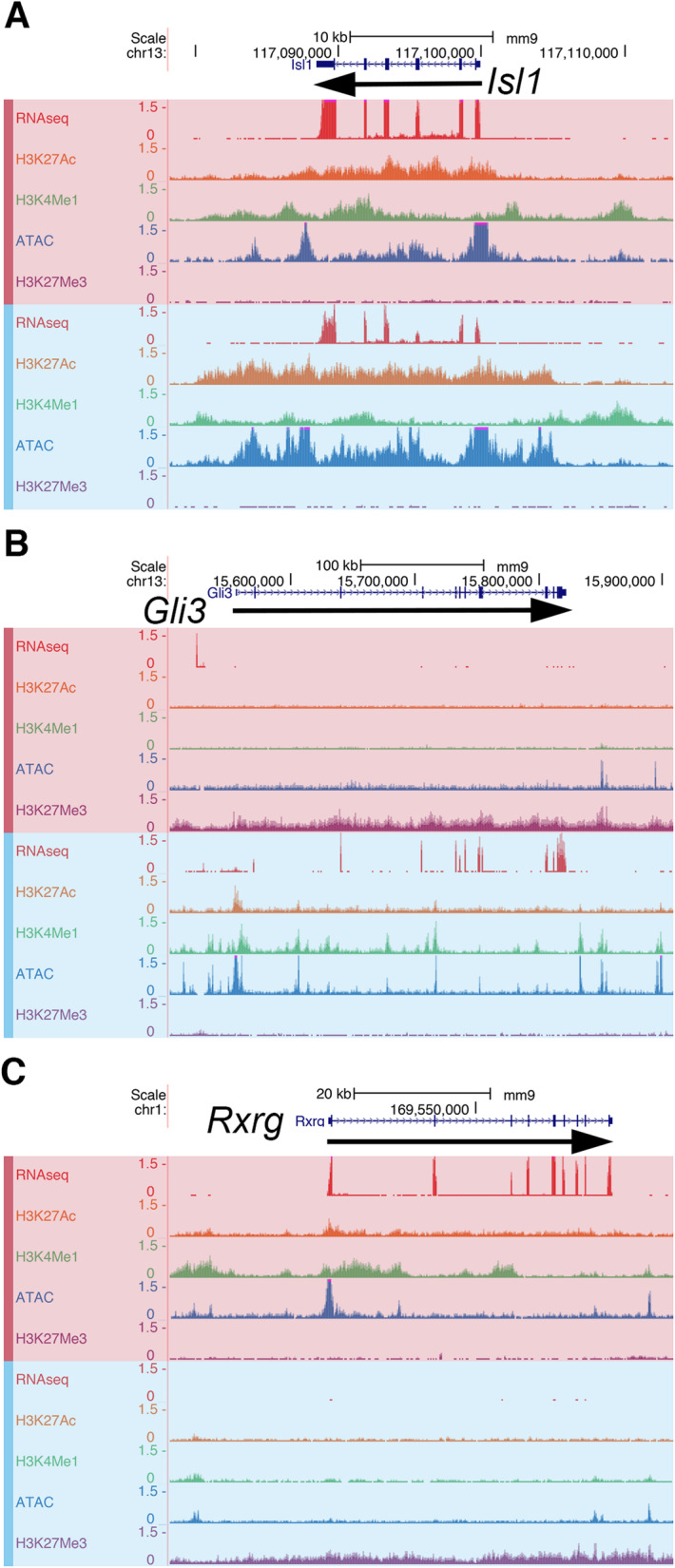
Epigenomic marks and expression for three key transcription factors. **a** *Isl1* encodes a key pituitary transcription factor that is expressed in both TαT1 (red) and GHF-T1 (blue) cell lines. Data from RNA-Seq, CUT&RUN for active chromatin marks (H3K27Ac and H3K4Me1), open chromatin (ATAC-seq), and CUT&RUN for repressive chromatin marks (H3K27Me3) is visualized for each cell line in genome browser tracks. **b** *Gli3* is in active chromatin and expressed in GHF-T1 cells. *Gli3* is not expressed in TαT1 cells, and the chromatin is inaccessible with repressive marks. **c** *Rxrg* is expressed and in active chromatin in TαT1 cells, while not expressed and bearing repressive marks in GHF-T1 cells. *N* = 2 for histone marks/cell line

To assess genome-wide changes in the chromatin landscape associated with thyrotrope differentiation, we performed Cleavage Under Target and Release using Nuclease (CUT&RUN) for three major histone marks: H3K27Ac, H3K4Me1, and H3K27Me3 [41]. The presence of both H3K27Ac and H3K4Me1 mark active enhancers, while H3K27Me3 marks repressed regions [42–44]. We also performed an Assay for Transposase-Accessible Chromatin with High-Throughput Sequencing (ATAC-seq), a method for profiling regions of accessible chromatin, which are often regulatory [45]. The results for three selected genes, *Isl1, Gli3,* and *Rxrg*, are shown in Fig. 2. We chose *Isl1* because of its important role in thyrotrope and gonadotrope fate [19], *Gli3* for its role in hypothalamic and pituitary development in humans, [17], and *Rxrg* because of its role in thyrotrope function and retinoic acid regulation of gene expression [46, 47]. Also, *Gli3* and *Rxrg* are the most and second-most differentially expressed transcription factors in the GHF-T1 and TαT1 cells, respectively. These data (called tracks) reveal that *Isl1* is expressed in both cell lines and has extensive H3K27Ac, H3K4Me1, and ATAC-seq signal across the locus, revealing putative active enhancers and areas of open chromatin. For example, the stretch of H3K4Me1 and H3K27Ac signal covering the last intron and penultimate exon of *Isl1* could be an enhancer (Fig. 2a).

We visualized the expression and chromatin architecture around genes that are differentially expressed in the precursor and differentiated cell lines. *Gli3* is strongly expressed in GHF-T1 cells and has many H3K27Ac, H3K4Me1, and ATAC-seq peaks, revealing active enhancers in areas of open chromatin (Fig. 2b). By contrast, *Gli3* is not expressed in TαT1 cells. The chromatin surrounding *Gli3* in the TαT1 cells is devoid of H3K27Ac, H3K4Me1, and ATAC-seq peaks and is covered with H3K27Me3, a mark of active repression. This shows that *Gli3* is actively repressed in the TαT1 cell line. Conversely, *Rxrg*, a gene whose deletion in mice is associated with thyroid hormone resistance [46], is highly expressed in TαT1 cells but not in GHF-T1 cells (Fig. 2c). Consistent with this, in TαT1 cells the *Rxrg* locus is decorated with H3K27Ac, H3K4Me1, and ATAC-seq peaks, whereas the GHF-T1 line has no such peaks, and shows active repression of *Rxrg*, with a broad H3K27Me3 signal. Additional tracks for selected genes that are expressed at similar levels, higher in GHFT1, or higher in TαT1 are presented in Supplemental Figures 2, 3 and 4, respectively.

We used ChromHMM to annotate different chromatin states based on H3K27Ac, H3K4Me1, H3K27Me3, and ATAC-seq signal [48]. Iterating over increasing numbers of possible states, we found that 11 states best captured the chromatin architecture within these two cell lines (Supplemental Figure 5). Of these states, two had both H3K4Me1 and H3K27Ac, predicting active enhancers. The difference between the two states was the presence or absence of an ATAC-seq signal, meaning one state represented open, active enhancers, while the other represented active enhancers in a more closed state.

### POU1F1 binding

**Fig. 3:**
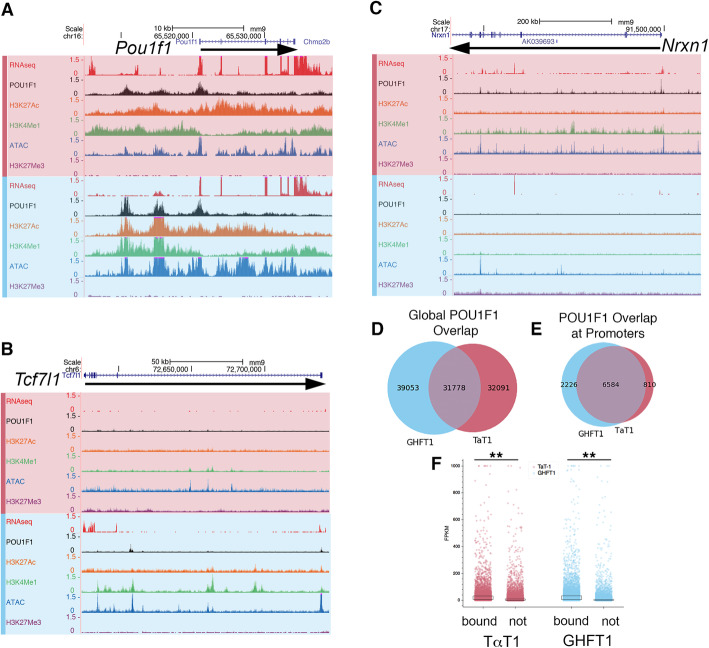
Comparison of POU1F1 binding sites in GHFT1 and TαT1 cells. **a** *Pou1f1* is expressed in both cell lines, contains active chromatin marks, and exhibits similar POU1F1 binding to enhancer elements. Data from RNA-seq, CUT&RUN for POU1F1 and active chromatin marks (H3K27Ac, H3K4Me1), ATAC-seq, and CUT&RUN for repressive marks (H3K27Me3) is visualized in genome browser tracks for TαT1 cells (red) and GHF-T1 (blue). **b** *Tcf7l1* is an example of a gene uniquely bound by POU1F1 and expressed in GHF-T1 cells. **c** *Nrxn1* is an example of a gene uniquely bound by POU1F1 and expressed in TαT1 cells. **d** A Venn diagram illustrating the number of shared and distinct POU1F1 binding sites throughout the genomes of GHF-T1 and TαT1 cells. **e** A Venn diagram showing the number of shared and distinct genes whose promoters and regulatory elements are bound by POU1F1 in GHF-T1 and TαT1 cells. **f** Scatter plot showing the FPKM of genes whose promoters are bound or not bound by POU1F1 in both TαT1 and GHF-T1 cells. In both TαT1 and GHF-T1 cells, genes whose promoters are bound by POU1F1 are more highly expressed. The boxed regions represent the middle quartiles, i.e., 25–75%. The significance is represented by asterisks (*p* values 4 × 10^− 114^ and 8 × 10^− 130^, respectively). *N* = 1 for POU1F1/cell line

We performed CUT&RUN for POU1F1 in both the GHF-T1 and TαT1 cell lines to identify similarities and differences in POU1F1 binding at these two stages of differentiation. We present three specific examples of POU1F1 binding. *Pou1f1* has two enhancers, a proximal (5.6 kb), early-stage enhancer bound by PROP1, and a distal (10 kb), late-stage enhancer. POU1F1 binds the distal enhancer and drives its own expression in an auto-regulatory fashion [49, 50]. In both cell lines, CUT&RUN shows extensive POU1F1 binding across the *Pou1f1* promoter-proximal region and both the early and late enhancers (Fig. 3a). The *Tcf7l1* gene is an example of elevated expression and preferential POU1F1 binding in GHF-T1 cells relative to TαT1 cells (Fig. 3b). TCF7L1 is important for hypothalamic-pituitary development and function in humans and mice [51]. Selective POU1F1 binding was detected at the *Nrxn1* promoter in TαT1 cells relative to GHFT1 cells, and *Nrxn1* expression increased from nearly zero in GHFT1 cells to 5 FPKM in TαT1 cells (Fig. 3c). *Nrxn1* is critical for proper synapse formation, but the role in pituitary is unknown [52].

Genome-wide analysis of POU1F1 binding in both cell lines revealed that the majority of binding is in putative enhancers and associated with higher levels of gene expression. There are a significant number of unique POU1F1 binding sites in each cell line: only one third of all POU1F1 binding sites are shared between the two lines (Fig. 3d). Only 15–16% of POU1F1 binding sites in GHF-T1 cells (10,980 out of 69,644) and TαT1 cells (9360 out of 63,036) are within 1 kb of a transcription start site (TSS), suggesting most POU1F1 binding is at enhancers. Consistent with this, nearly 70% of genes whose promoters are bound by POU1F1 in the differentiated line are also bound by POU1F1 in the precursor line (Fig. 3e). We found that POU1F1 binding is associated with higher levels of gene expression in both cell lines (Fig. 3f).

**Fig. 4:**
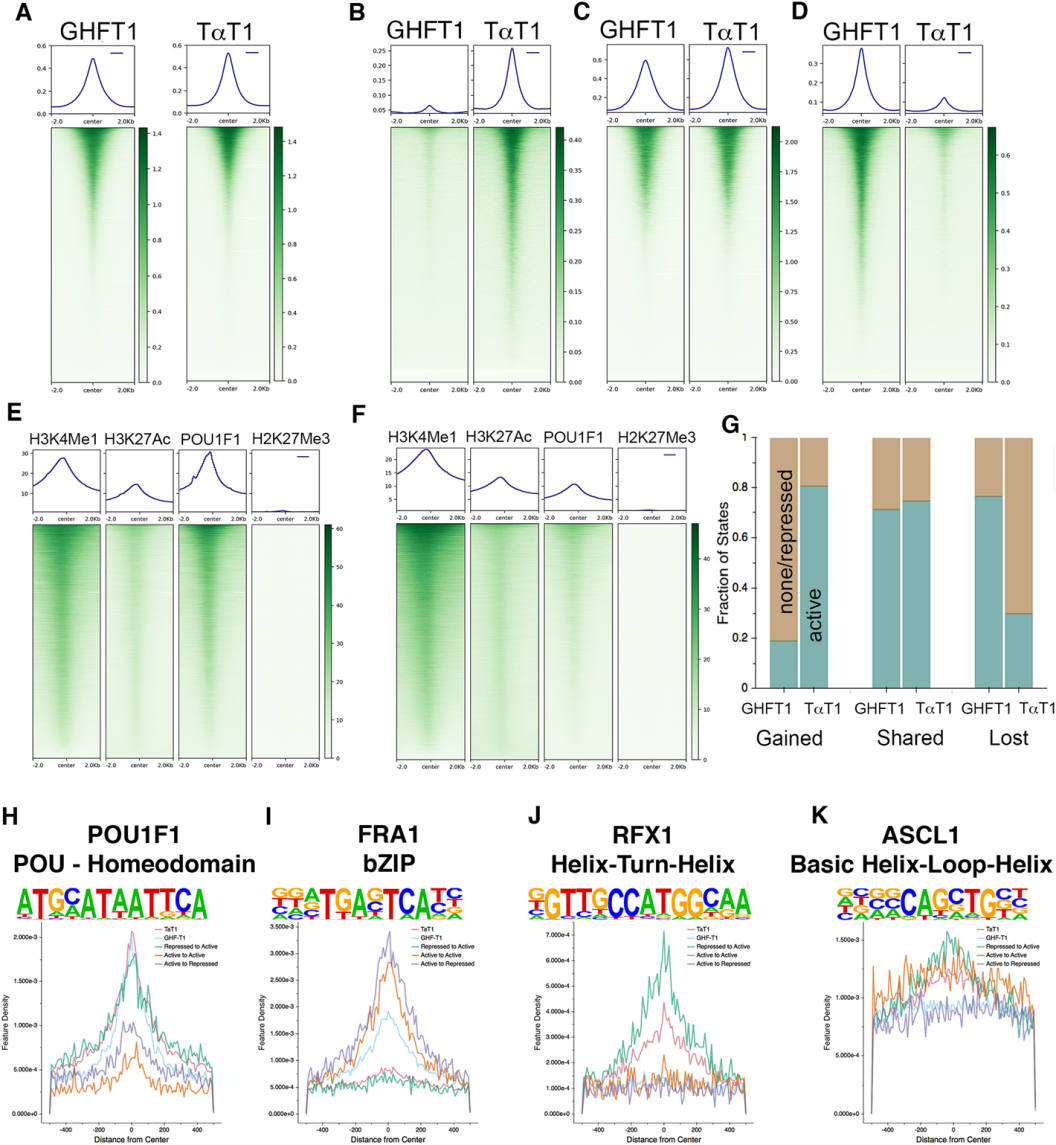
Comparison of open chromatin between GHF-T1 and TαT1 cells and prediction of transcription factor binding. **a** ATAC-seq signal at POU1F1 binding sites in GHFT1 and TαT1 cells. **b** ATAC-seq signal at POU1F1 binding sites that are specific to TαT1 cells. **c** ATAC-seq signal at POU1F1 binding sites that are shared between Tα T1 and GHF-T1 cells. **d** ATAC-seq signal at POU1F1 binding sites that are specific to GHF-T1 cells. **e** POU1F1 signal at enhancers in GHFT1 cells. **f** POU1F1 signal at enhancers in TαT1 cells. **g** The composition of active (teal) chromatin states vs repressed or unmarked chromatin states (brown) for POU1F1 binding sites in GHF-T1 and TαT1 cells is compared. Active states are defined as states 1–6 (Supplemental Figure 5). TαT1-specific sites (gained in differentiation, left) are mostly active in TαT1 cells, while shared sites (center) have equivalent active states. GHF-T1-specific sites (lost in differentiation, right) are mostly active in GHFT1 cells. **h** Density of POU1F1 motifs across POU1F1 binding sites in GHF-T1 cells (blue), TαT1 cells (red), at TαT1-specific POU1F1 binding sites that are repressed in GHF-T1 cells and active in TαT1 cells (repressed to active, green), POU1F1 binding sites that are shared in GHF-T1 and TαT1 cells that are active in both (active to active, orange), and POU1F1 binding sites that are specific to GHF-T1, and are in an active state in GHF-T1 cells and a repressed state in TαT1 cells (active to repressed, purple). **i** Similar analysis as **h**, for the bZIP transcription factor sites, like FRA1. **j** Similar analysis as **h**, for the HTH transcription factor sites, like RFX1. **k** Similar analysis as **h**, for bHLH transcription factor sites, like ASCL1. Supplemental Figure 6 shows more motifs

POU1F1 binding is associated with higher ATAC-seq signals in GHF-T1 cells than in TαT1 cells (Fig. 4a). As expected, sites of POU1F1 binding specific to TαT1 cells are more open in TαT1 cells (Fig. 4b), sites of POU1F1 binding specific to GHF-T1 cells are more open in GHF-T1 cells (Fig. 4c), and shared POU1F1 sites have similar signatures of open chromatin in both cell lines (Fig. 4d).

Active enhancers (states containing both H3K27Ac and H3KMe1 in ChromHMM) are heavily enriched for POU1F1 binding in both cell lines (Fig. 4e, f). GHF-T1 enhancers appear to have greater POU1F1 binding than do TαT1 enhancers. POU1F1 binding that is specific to TαT1 cells is associated with active chromatin states in TαT1, but it is less so in GHF-T1 cells (Fig. 4g). Similarly, GHF-T1-specific POU1F1 binding is broadly associated with active chromatin states in GHF-T1 cells, but it less so in TαT1 cells. Sites bound by POU1F1 in both cell types have similarly active chromatin states, as expected.

To identify transcription factors that may be associated with differential POU1F1 binding between the cell lines, we analyzed the chromatin states associated with shared and unique POU1F1 binding sites and screened these for binding motifs. We classified genomic sites that had POU1F1 binding exclusively in TαT1 cells, were in active states in the TαT1 cells, and were in repressed states in GHF-T1 cells (repressed to active), POU1F1 binding sites that are shared between GHF-T1 and TαT1 and are in similarly active chromatin in both (Active to Active), and GHF-T1-specific POU1F1 binding sites that are in active chromatin in GHF-T1 sites and are repressed in TαT1 cells (labeled active to repressed). This revealed an expected enrichment in POU1F1 motif density at the center of POU1F1 sites in both GHF-T1 and TαT1 cells (Fig. 4h). There was a striking enrichment of bZIP motifs at the center of GHF-T1-associated POU1F1 binding sites (Fig. 4i), suggesting that bZIP factors influence POU1F1 binding in progenitors.

Interestingly, there was remarkable helix-turn-helix motif density at the center of TαT1 POU1F1 binding sites, and even more so at repressed to active sites (Fig. 4j). Similarly, there was increased bHLH motif density at repressed to active sites (Fig. 4k). These data suggest that HTH and bHLH factors mediate POU1F1 binding to novel sites in thyrotropes.

### Stretch enhancers

**Fig. 5:**
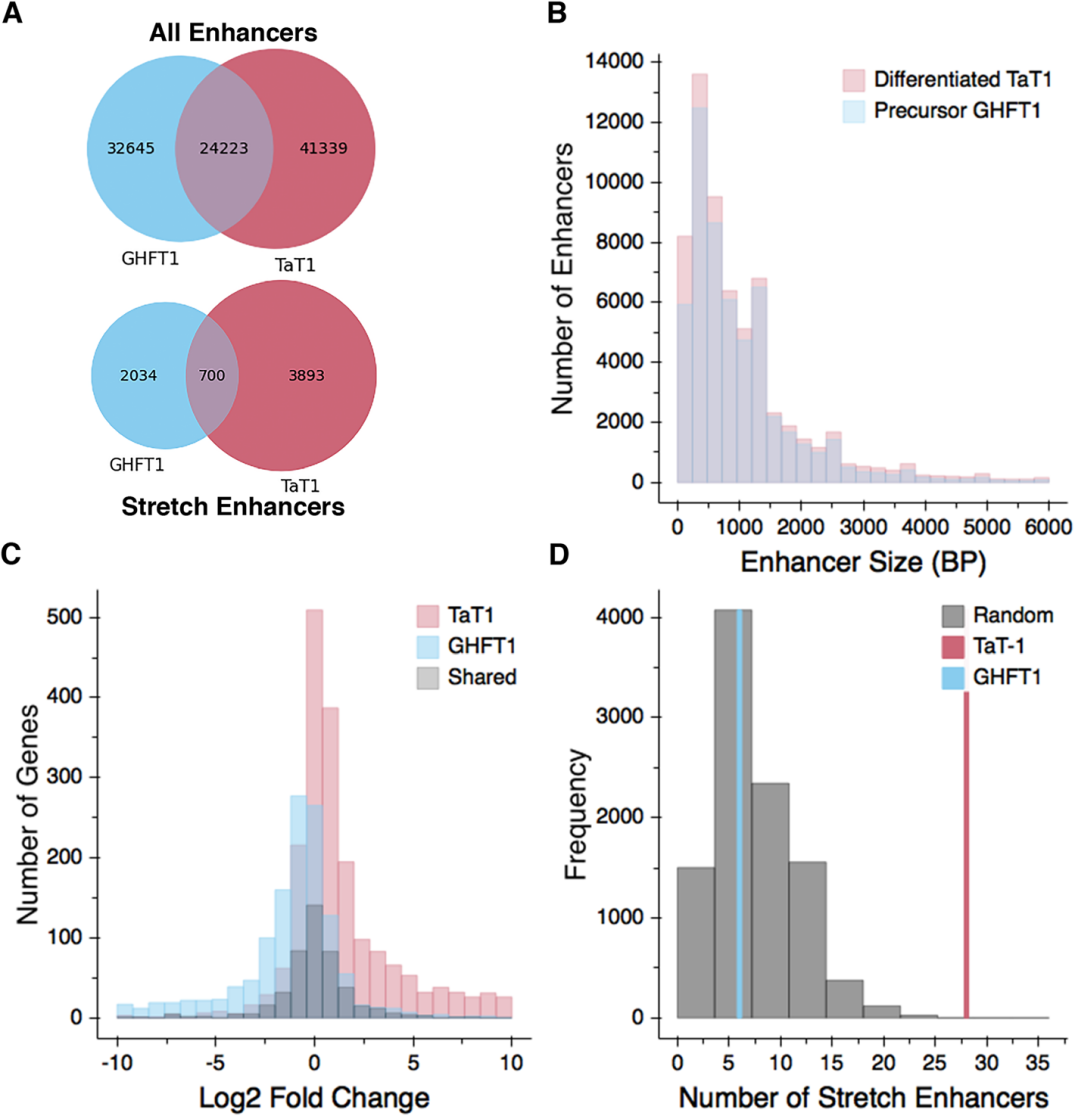
Comparison of putative enhancers in GHF-T1 and TαT1 cells. **a** A Venn diagram showing the number of shared and distinct enhancers in GHF-T1 and TαT1 cells (top). A Venn diagram showing the number of shared and distinct stretch enhancers in GHF-T1 and TαT1 cells (bottom). **b** A histogram showing the distribution of enhancer sizes in GHF-T1 (in blue) and TαT1 cells (in red). **c** A histogram showing the log 2-fold-change in expression of the genes that are closest to GHF-T1-specific stretch enhancers (blue), TαT1-specific stretch enhancers (red), and shared stretch enhancers (black). **d** Number of TαT1 (red) and GHF-T1 (blue) stretch enhancers within 100 kb (50 kb upstream or downstream) of the TSS of 25 genes important for thyrotrope function (Supplemental Table 3). To evaluate the significance of the difference, 25 genes were randomly selected, normalized for gene expression, and assessed for stretch enhancers. This process was repeated in 10,000 iterations and displayed as a histogram (gray)

Twenty-four percent of the putative enhancers that we identified were in open chromatin in both the precursor and differentiated cell lines, as defined by at least 25% bidirectional overlap (Fig. 5a). There were 15% more areas of open chromatin at putative enhancers in the differentiated, thyrotrope state than the precursor state. The distribution of enhancer sizes was very similar between the two cell lines (Fig. 5b). Enhancers larger than 3 kb in length, called stretch enhancers, represent 5–10% of all enhancers, are typically cell-type specific and often enriched in disease-associated areas [53, 54]. Stretch enhancers represent 4.9% of the enhancer population in the precursor cell lineage and 7.1% of the enhancer population in the differentiated thyrotrope population. This is within the expected fraction, and the increased abundance in TαT1 cells is consistent with their more differentiated state. While GHF-T1 and TαT1 cells share 24% of all enhancers, only 10% of stretch enhancers are shared between the two cell types (Fig. 5a).

We compared the expression of the closest gene to each stretch enhancer and found that expression was highly cell-type specific. Genes closest to precursor stretch enhancers were more highly expressed in the precursor cell line, whereas the genes closest to thyrotrope stretch enhancers were expressed at higher levels in the thyrotrope cell line (Fig. 5c). Genes closest to shared stretch enhancers had similar gene expression in both cell lines.

We sought to determine whether genes associated with thyrotrope function in both mouse and human were closer to stretch enhancers. We generated a list of 25 candidate genes associated with thyrotrope differentiation and/or function (Supplemental Table 3), and we found that the TSSs of all of these genes were within 100 kb of 28 TαT1 stretch enhancers and only 6 GHF-T1 stretch enhancers (Fig. 5d). To determine whether this result was significant, we randomly selected 25 genes 10,000 times, ensuring the genes had similar expression levels, and we counted the number of stretch enhancers within 100 kb of the transcription start site of those randomly selected genes. The randomly selected genes were within 100 kb of 28 TαT1 stretch enhancers only two times out of 10,000, yielding an empirical *p* value of 0.0002, which confirms the enrichment of TαT1 stretch enhancers at thyrotrope-signature genes.

To probe the potential value of these data for application to human disease studies, we mapped the mouse enhancers in TαT1 and GHF-T1 cells from the mm9 genome onto the human genome, hg19*.* Because ~ 90% of GWAS SNPs are intronic or intergenic, and stretch enhancers are heavily enriched for disease SNPs, we expected to implicate thyrotropes in disease phenotypes by uncovering enrichment of disease SNPs in TαT1 stretch enhancers [4, 53]. We used GARFIELD to measure the enrichment of these SNPs in GHF-T1 and TαT1 and stretch enhancers, while accounting for linkage disequilibrium, minor allele frequency, and distance to TSS [55]. We compared their enrichment to stretch enhancers found in heterologous cell lines including but not limited to Islet cells, GM12878 (human B-lymphocyte cells), and K562 (human myelogenous leukemia cells) cells [53]. Supplemental Figure 7 shows the enrichment odds ratios for all GWAS studies and cell line stretch enhancers, as well as their *p* values. The study that exhibited the greatest enrichment of SNPs in TαT1 stretch enhancers (odds ratio of 4.7) was from GWAS done on the neuroticism sub-phenotype of feeling miserable [56]. While the significance of this is uncertain, untreated hypothyroidism can be associated with fatigue and depression.

### In vitro validation of enhancers

**Fig. 6:**
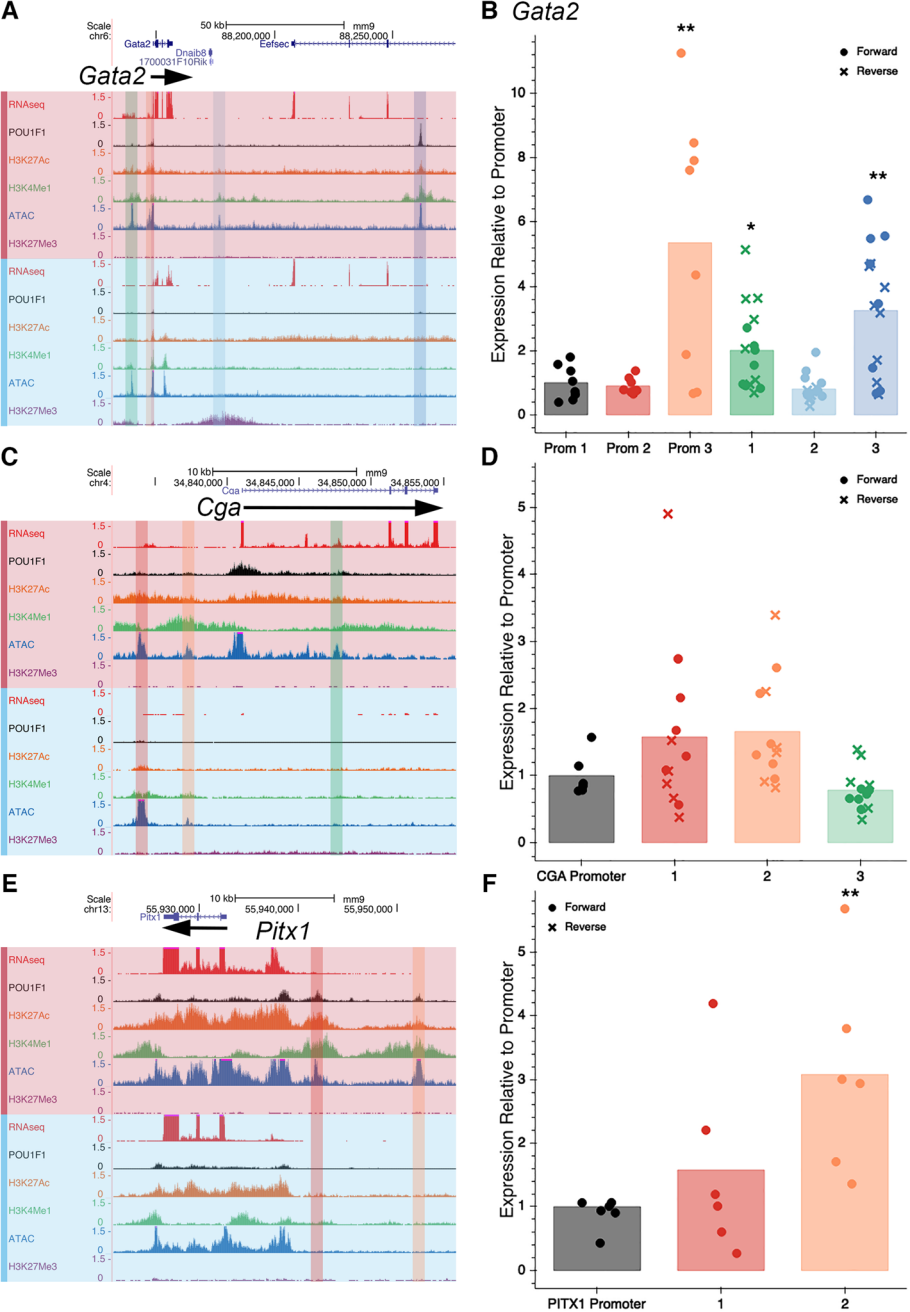
Functional testing of putative enhancers by transient transfection. **a** Tracks for RNA-seq, POU1F1, H3K27Ac, H3K4Me1, ATAC-seq, and H3K27Me3 are shown for *Gata2* in TαT1 (red) and GHF-T1 (blue) cells. The putative enhancer regions cloned for the luciferase assay are highlighted in red, orange, green, light blue, and dark blue. **b** The level of luciferase activity of each element relative to the smallest promoter region is shown. Prom 1, 2, and 3 represent the *Gata2* promoter with 0.2, 0.9, and 2.8 kb of 5′ flanking region, respectively. 1, 2, and 3 represent the three similarly highlighted elements in **a** tested in both the forward (circles) and reverse (x’s) orientation upstream of the 0.2 kb *Gata2* promoter. The significance was evaluated with a two-sided *t*-test and indicated with asterisks where *p* value < 0.05 = * and *p* value < 0.01 = **. **c** Same tracks as in **a**, at the *Cga* locus, where elements tested are highlighted. **d** Level of luciferase activity of each element, color-coordinated with the highlighted elements in **c** in both the forward (circles) and reverse (x’s) orientation. **e** Same tracks as in **a** at the *Pitx1* locus, where elements tested are highlighted. **f** Level of luciferase activity of each element, color-coordinated with the highlighted elements in **f**. Elements were tested only in the forward orientation. *N* = 6 replicates/reporter gene

We sought to test putative enhancers of a sampling of thyrotrope-signature genes, namely *Gata2*, *Cga*, and *Pitx1*, by transient transfection of TαT1 cells. We identified putative regulatory elements as regions with significantly enriched ATAC-seq signals near these genes. The promoter-proximal sequences of each gene were amplified from genomic DNA and fused to a luciferase reporter gene. Putative regulatory elements were amplified from genomic DNA and cloned in both the forward and reverse orientation upstream of the promoter-proximal region. The transfection efficiency of TαT1 cells is low (~ 20%), and the results vary, likely due to poor adherence of cells to the plate. Thus, all experiments were performed with six replicates. We detected strong enhancer elements for *Gata2* and *Pitx1* (Fig. 6). The position (genome coordinates) of cloned promoters and elements is presented in Supplemental Table 4.

*Gata2* is implicated in *Tshb* transcription and proper thyrotrope function [13, 18, 21]. *Gata2* has two promoters located upstream of a noncoding exon [57]. The more distal promoter is located ~ 5 kb upstream of the more proximal promoter, and it drives expression in Sca-1+/c-kit+ hematopoietic progenitor cells, while the downstream promoter drives *Gata2* expression in most other tissues. The RNA-seq data revealed that the downstream promoter is the only one utilized in TαT1 and GHF-T1 cells. *Gata2* expression is five-fold higher in TαT1 cells than in GHF-T1 cells and there is a larger area of accessible chromatin upstream of *Gata2* in the TαT1 cells (~ 2.8 kb vs 0.9 kb, Fig. 6a). There is no difference in activity between the 0.2 kb and 0.9 kb promoter-proximal region in TαT1 cells, but the larger, 2.8 kb promoter-proximal region of *Gata2* stimulated luciferase activity 5-fold (*p* value = 0.009). This indicates the presence of enhancer elements between 0.9 and 2.8 kb of the common TSS for *Gata2*. We tested three distal elements, fusing them with the smallest, 0.2 kb *Gata2* promoter construct. An element ~ 100 kb 3′ of the common *Gata2* promoter had significant POU1F1 binding and drove the highest levels of luciferase expression (3-fold increase, *p* value = 0.005). Thus, we identified two enhancer elements for *Gata2* expression in thyrotropes, one in the proximal promoter region, within 2.8 kb of the TSS, and a more distal one, approximately 110 kb downstream.

*Cga* is the alpha-subunit of TSH, and the gonadotropins, FSH and LH, and it is expressed in both thyrotropes and gonadotropes. We tested three *Cga* enhancer elements, and two appeared to have some activity, although they did not reach statistical significance. The element ~ 7 kb upstream of *Cga* increased luciferase activity 1.5-fold, and it has not been previously described. The element located 4.6 kb upstream of the *Cga* gene increased luciferase activity 1.6-fold and approached statistical significance (*p* value = 0.07). This element is sufficient for developmental activation, cell type-specific expression, and hormonal regulation in transgenic mice (Fig. 6b) [16, 58, 59].

*Pitx1* has a role in *Pomc* expression and hindlimb formation in mice and humans [60, 61]. *Pitx1* and the related *Pitx2* gene drive early pituitary development and are expressed in thyrotropes and gonadotropes [24]. Mice with a pituitary-specific disruption of *Pitx2* exhibit elevated *Pitx1* expression in response to induced hypothyroidism, suggesting functional compensation [22]. The *Pitx1* regulatory landscape extends over 400 kb and includes a pituitary enhancer 110 kb upstream [62]. ATAC-seq signatures revealed two previously undescribed thyrotrope-specific regions of open chromatin 9 and 19 kb upstream of the *Pitx1* transcription start site (Fig. 6c). The proximal element did not have statistically significant enhancer activity. The distal element, however, increased luciferase activity 3-fold (*p* value = 0.009).

### Discovery of a novel TSH β-subunit enhancer

**Fig. 7:**
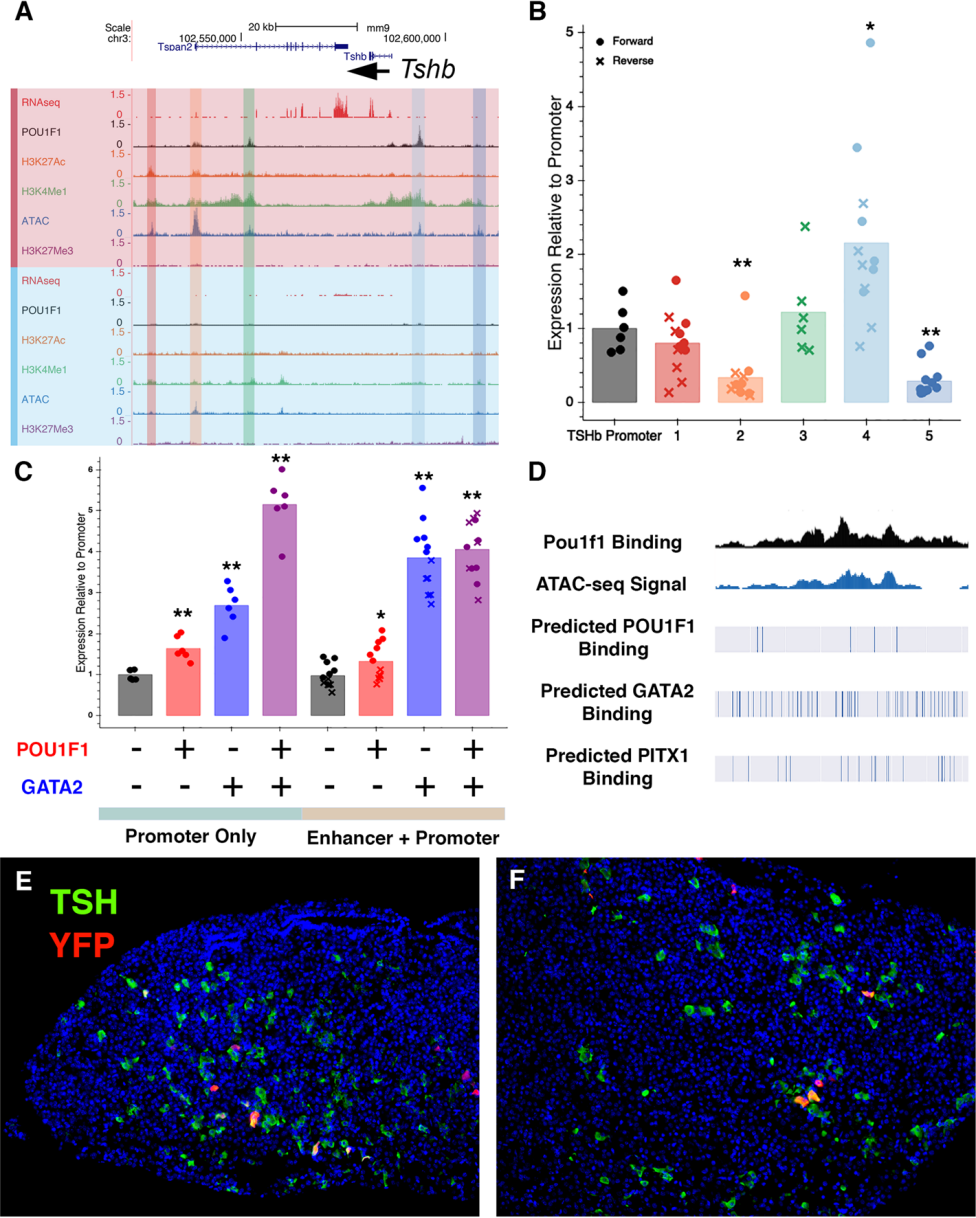
Evaluation of *Tshb* regulatory elements in transfection and transgenic mice. **a** Tracks illustrating the results of RNA-seq, CUT&RUN for POU1F1, H3K27Ac, and H3K4Me1, ATAC-seq, and CUT&RUN for H3K27Me3 are shown for the *Tshb* locus in TαT1 cells (red) and GHF-T1 cells (blue). Elements that were tested functionally are highlighted. The significance is indicated with asterisks: *p* value < 0.05 = *; *p* value < 0.01 = **. **b** TαT1 cells were transfected with a 438 bp *Tshb* promoter-proximal element fused to luciferase and putative regulatory elements in the forward (filled circles) or reverse (x) orientation. Luciferase activity for each construct is normalized to the promoter-only luciferase activity and significant differences from promoter alone are indicated with asterisks (*p* < 0.05 = * and *p* < 0.01 = **). **c** Heterologous CV1 cells were transfected with the *Tshb* promoter-only construct or the promoter plus element 4 construct, along with expression vectors for POU1F1 and/or GATA2 as indicated by + or −. Significant differences from the reporter gene alone are indicated with asterisks (*p* < 0.05 = * and *p* < 0.01 = **). The values obtained for both constructs with POU1F1 or GATA2 expression vectors were significantly different, *p* < 0.01. **d** Genome browser track illustrating element 4 (1.4 kb located 6.6–7.7 kb upstream of the *Tshb* transcription start site) with experimentally determined sites for POU1F1 binding and ATAC-seq, and predicted binding sites for POU1F1, GATA2, and PITX1 that reach a confidence level of at least 0.8 in JASPAR. An extended list of binding motifs predicted in Element 4 is presented is in Supplemental Table 5. **e** A section of the pituitary gland from transgenic founder 399 was co-immunostaining for YFP (red) and TSHB (green), revealing overlap in expression (yellow). Nuclei are stained with DAPI (blue). **f** Same as **e**, in founder mouse 423. For transfections, *N* = 6/reporter gene

A bacterial artificial chromosome clone containing *Tshb* and 150 kb of surrounding DNA sequence was sufficient to drive expression in thyrotropes of transgenic mice [22], but there is no information about the location of key regulatory elements within this region. In fact, multiple efforts to drive expression in transgenic mice with smaller constructs were unsuccessful [63]. We sought to leverage the information we have about the chromatin states in the TαT1 cells to identify elements sufficient for *Tshb* expression in mice. Knowing that the 150 kb BAC was sufficient to drive expression in thyrotropes, we limited our search to this region and found five areas with high ATAC-seq signal in TαT1 cells (Fig. 7a). We tested each of these elements both in the forward and reverse orientation fused to a 438 bp *Tshb* promoter-proximal region driving luciferase expression (Fig. 7b, genomic coordinates of the cloned promoter and putative enhancer elements are in Supplemental Table 4). We discovered that a 1.4 kb element located 7.7–6.6 kb upstream of the *Tshb* transcription start site drove significant levels of luciferase (hereafter named element 4). Element 4 had extensive ATAC-seq signal and H3K4Me1 and POU1F1 binding, consistent with the observation that POU1F1 is important for *Tshb* expression. We tested the ability of GATA2 and POU1F1 to activate *Tshb* promoter alone and in conjunction with element 4 in heterologous CV1 cells. GATA2 and POU1F1 independently cause modest increases in *Tshb*-luc reporter gene expression (2.7-fold and 1.6-fold respectively), and together they drive higher expression (5-fold, Fig. 7c), similar to previous reports [13]. In the presence of element 4, POU1F1 has very modest effects on reporter gene expression (1.3-fold increase), but GATA2 has a strong effect (4.3-fold increase). GATA2 and POU1F1 do not have an obvious additive effect on element 4 reporter activity (4.4-fold increase) in contrast to the promoter-proximal region. This suggests that GATA2 is a powerful regulator of *Tshb* expression through interaction with element 4. To determine which other factors may be binding Element 4, we checked for the presence of over 1000 Jaspar motifs within element 4 at an 80% threshold. We found extensive predicted GATA2 and PITX1 binding (Fig. 7d). A more complete list of predicted binding factors is presented in Supplemental Table 5.

We tested whether element 4 was sufficient to drive expression in transgenic mice by placing it in front of the *Tshb* promoter and a YFP reporter gene. This construct was injected into fertilized eggs that were subsequently transferred to pseudopregnant foster mothers. We dissected the pituitaries of eleven founder transgenic mice at 4 weeks of age and examined the expression of YFP using immunohistochemistry (Fig. 7e, f). Forty-five percent (5/11) exhibited transgene expression in the pituitary gland: three founders had low levels of YFP expression, and two founder mice had higher levels of YFP activity. This contrasts with our previous transgenic analyses using − 1.1 kb to + 620 bp of *Tshb* that yielded no expression (0/3 lines), and a larger construct containing − 6 kb to + 43 bp that yielded no expression (0/4 lines), even after 10 days of treatment with propothiouracil, which would be expected to increase *Tshb* transcription at least ten-fold [63]. We quantified expression in two element 4 transgenics. 87% of YFP-positive cells (*N* = 63/76) were also positive for TSH in founder 399, indicating high specificity for thyrotropes, and 6% of the transgenic thyrotropes were also positive for YFP (63/872), signifying low penetrance of expression. In total, 31% of YFP-positive cells (8/26) were positive for TSH in founder 423, and 3% of the transgenic thyrotropes were also positive for YFP (8/270). This suggests that element 4 contributes to *Tshb* expression, although other elements are required for higher penetrance expression. This serves as a proof of the principle that combined transcriptome and epigenome data can be valuable for identifying enhancer elements that function in developmentally specific cell lines and intact animals.

## Discussion

Our work builds on the ENCODE effort to discover regulatory elements in diverse tissues. This represents the first systematic characterization of the epigenome and transcriptome of a thyrotrope-like cell line, providing insight into the changes that are associated with the differentiation of committed POU1F1 pituitary cells into thyrotropes. POU1F1 has a similar binding profile at promoters in the two cell lines, but there are novel binding sites at enhancers in each cell line that are associated with striking shifts in chromatin states. Global analysis of positive histone marks (H3K27Ac and H3K4Me1) revealed putative active enhancers in the two cell lines. We demonstrated that many of the enhancers surrounding thyrotrope-signature genes drive expression in a thyrotrope cell line. Furthermore, an enhancer element upstream of *Tshb* was sufficient to drive expression in some thyrotropes in transgenic mice. The transcriptomic, epigenomic, and POU1F1 binding data here contributes significantly to our understanding of thyrotrope cell specification, which are the key cells for regulation of thyroid gland development and growth and metabolism.

This is the first deep expression profiling reported for GHF-T1 and TαT1 cells. The TαT1 cells have been used extensively as a thyrotrope-like cell to study the molecular mechanisms of hypothalamic input and thyroid hormone feedback on *Tshb* expression, and remarkably, they also respond to retinoids and secrete TSH in response to diurnal cues [28–30]. In total, 92% of the genes we predicted would be expressed in a thyrotrope-like cell were detected in TαT1 cells at > 1 FPKM (Supplemental Table 3). Recent single-cell gene expression studies of pituitary glands from adult rodents and developing human fetuses have revealed expected and novel genes enriched in thyrotropes, including *Tshb, Trhr, Dio2, Pcsk2, Dpp10, Sox11, Nrg4,* and others [64–66]. The majority (77%, *N* = 35) of thyrotrope-enriched genes identified by single-cell sequencing were expressed at levels > 1 FPKM in TαT1 cells, and 96% of those genes exhibited elevated expression in TαT1 cells relative to GHFT1 (*N* = 27) (Supplemental Figure 9, Supplemental Table 6). Thus, the gene expression data confirm the strong relationship between TαT1 cells and thyrotropes in vivo.

As hormone-producing cells mature, they ramp up translational machinery for robust hormone production, and CREB3L2 is a master regulator of this process in the pituitary corticotropes [67]. While *Creb3l2* is not highly expressed in TαT1 cells, *Creb3l1* is expressed nearly 30-fold higher in TαT1 cells than in GHF-T1 cells (172.5 FPKM vs. 5.9 FPKM). It is possible thyrotropes use a similar mechanism of increasing translation to meet the demand for thyrotropin. Consistent with this, *Creb3l1* is upregulated in a model of thyrotrope adenoma [68].

Pituitary endocrine cells are electrically excitable, and voltage-gated calcium influx is the major trigger for hormone secretion [37]. G protein-coupled receptors, ion channels, and hormones all are considered components of cellular identity. For example, thyrotropes have unique electrical activity relative to other pituitary hormone-producing cell types. TαT1 cells exhibit a bursting pattern of action potentials that are affected by exposure to TRH and thyroid hormone, but the nature of the ion channels regulating TSH secretion is not understood [69, 70]. The involvement of ion channels in excitation-secretion coupling is an area of active study. The hypothalamic factors CRH, TRH, GHRH, and somatostatin have an effect on electrical activity in corticotropes, lactotropes, and somatotropes. Thyrotropes have not been well-studied in this regard. Our study provides evidence for the acquisition of ion channel gene expression as progenitors adopt the thyrotrope fate. Voltage-gated potassium channels *Kcnc3*, *Kcnq2*, *Kcnk1*, and *Kcnk2* were highly expressed in TαT1 cells. Interestingly, *KCNQ1* missense mutations cause growth hormone deficiency [71]. We also found that sodium channel genes are more highly expressed in two cell types that express hormones, Pit1-triple and TαT1, than in lines that do not express hormones. The calcium channel CACNA1G, a low-voltage activated, T-type channel, was highly elevated in TαT1 cells. These observations suggest that ion channel expression may be acquired as cells begin to differentiate. Additional studies may be necessary to determine whether they are pruned during maturation [37]. Knowing which ion channels are expressed in thyrotrope cells is the first step in understanding the mechanism whereby TRH stimulates TSH release in a pulsatile manner and according to the appropriate diurnal rhythm.

Several transcription factors had higher levels of expression in TαT1 cells relative to GHFT1, including ISL1, RXRG, and LHX3. Increased expression of *Isl1* and *Rxrg* was expected because pituitary-specific deletion of *Isl1* causes reduced thyrotrope differentiation [19], and several lines of evidence support a role for *Rxrg.* RXRG suppresses serum TSH levels and *Tshb* transcription, *Rxrg-*deficient mice have central resistance to thyroid hormone, and loss of retinoic acid signaling suppresses thyrotrope differentiation [46, 47]. *Isl1* had extensive POU1F1 binding across the 1 MB region surrounding it. *Lhx3* expression is detectable at e9.5 in the mouse pituitary placode and expression persists though adulthood [72]. Thus, we expected to detect *Lhx3* transcripts in all pituitary cell lines. *Lhx3* transcripts were nearly undetectable in Pit1-zero cells and in the precursor GHF-T1 lineage, but transcripts were higher in Pit1-triple (1.6 FPKM) and highest in TαT1 cells (44.2 FPKM). Recently, an SV40-transformed pituitary precursor cell line was developed that expresses the stem cell marker SOX2 but not LHX3 [73]. There may be dynamic changes in *Lhx3* expression during development that has not been documented.

SHH signaling is critical for establishing the pituitary placode and induction of *Lhx3* expression [34]. The GHFT1 precursor lineage expressed *Gli2* and *Gli3*, which are downstream targets of SHH, at higher levels than TαT1 cells, suggestive of active SHH signaling. *Gli2* and *Gli3* promoters are associated with extensive H3K4Me1 and H3K27Ac, and active enhancers can be found upstream, downstream, and within their introns. By contrast, *Gli2* and *Gli3* have broad stretches of H3K27Me3 in the TαT1 line, a mark of active repression. The active expression of these elements in GHF-T1 cells underline how well these cell types represent the early pituitary state and suggest they could be for valuable for identifying GLI target genes that underlie pituitary developmental abnormalities [17].

POU1F1 is critical for development of thyrotropes, somatotropes, and lactotropes, and it likely interacts with other factors that specify the three different cell fates. Motif analysis near unique POU1F1 binding sites suggests which other factors may be involved. POU1F1 binding is associated with the homeodomain consensus binding motif, and sites of TαT1-specific POU1F1 binding that are repressed in GHF-T1 cells and active in TαT1 cells are heavily enriched for bHLH and HTH motifs. This raises the possibility that bHLH and HTH factors pioneer the binding of POU1F1 which then activates thyrotrope-specific expression.

Members of the RFX family of transcription factors are attractive candidates for interaction with POU1F1 to drive thyrotrope fate. These HTH factors contain DNA binding and heterodimerization domains and regulate cell fate in many organ systems, including the pancreatic islets and the sensory cells of the inner ear [74, 75]. They interact with other POU and SIX factors to direct fate. Several members of the gene family are expressed in both GHF-T1 and TαT1 cells and in pituitary development between e12.5 and e14.5, a time when progenitors leave the cell cycle and initiate differentiation [76]. Future studies will be necessary to define the role of these genes in pituitary development.

The expression of bHLH factor genes *Ascl1* (*Mash1*) and *NeuroD4* (*Math3*) are elevated in TαT1 cells. However, thyrotrope commitment is normal in *Ascl1* knockout and in triple knockout mice deficient in *Ascl1, Neurod4,* and *Neurod1.* These bHLH activating factors have overlapping functions in promoting somatotrope, gonadotrope, and corticotrope development [39, 40, 77, 78]. They promote pituitary stem cell exit from the cell cycle and act as selectors of cell fate, as triple knockout mice have more SOX2-positive cells and more lactotropes. bHLH factors play important roles in neuronal differentiation. For example, the BAM factors ASCL1, BRN2, and MYT1L are sufficient to transdifferentiate mouse embryonic fibroblasts (MEFs) into neurons. The Zn finger transcription factor MYT1L is highly upregulated in TαT1 cells, and although the POU factor *Brn2* is barely expressed in TαT1 cells, POU1F1 is highly expressed. Thus, it is possible that ASCL1 acts in concert with MYT1L and POU1F1 to drive thyrotrope fate.

The top three most highly expressed bHLH factors in both of pituitary cell lines are the repressive bHLH factors in the ID family. The role of these genes in pituitary development has not been studied, but they may be important in regulating progenitor differentiation and cell fate selection. For example, a proper balance of activating and repressive bHLH factors is critical for cortical development [79]. Repressive ID and Hes factors are expressed in cortical progenitors, and induction of key activating bHLH factors drives these progenitors to differentiate. Astrocytes, however, require continued repressive bHLH factor expression. The interplay of active and repressive bHLH factors in pituitary development is likely complex.

## Conclusion

This work represents the first thorough characterization of the epigenome and transcriptome in *Pou1f1* lineage progenitors and thyrotropes. We used this genome-wide catalog and tested enhancer function of elements in genes encoding thyrotropin, *Cga* and *Tshb*, the receptor for the hypothalamic releasing hormone that regulates thyrotropin, *Trhr*, and two crucial transcription factors, *Gata2* and *Pitx1*. This provides proof of the principle that the catalog is valuable for dissecting gene regulation. In addition, we demonstrate that the *Tshb* enhancer element is sufficient for expression in thyrotropes in transgenic mice and is directly regulated by GATA2. We discovered that unique POU1F1 binding sites are associated with bZIP factor binding motifs in *Pou1f1* lineage progenitors and bHLH or bHTH binding motifs in thyrotropes. This suggests candidate gene families for regulating thyrotrope differentiation. Of the more than 30 known genes that are mutated in patients with hypopituitarism, 18 are transcription factors that regulate pituitary development and cell specification, indicating the clinical importance of this field of study [80]. The overwhelming majority of patients with hypopituitarism have no molecular diagnosis, suggesting additional genes remain to be discovered [17]. We provide a rich selection of candidate transcription factors that are differentially expressed in progenitors and thyrotropes. Amongst the top 40 of these, 9 are already implicated in pituitary development and disease. Future analysis of the remaining 31 candidates may uncover additional disease genes.

## Methods

### Cell culture and transfection

GHF-T1 and TαT1 cells were provided by Dr. Pamela Mellon at University of California San Diego and grown on uncoated 100-mm dishes and Matrigel-coated 60-mm dishes, respectively. They were grown in DMEM (Gibco, 11995-065) with 10% fetal bovine serum (Corning, 35016CV) and 1% penicillin streptomycin (Sigma-Aldrich P4333). Cells were split 1:10 once they achieved 80% confluence. Six replicates of TαT1 cells were transfected using FuGENE 6 with a 3:1 transfection reagent/DNA ratio. Cells were collected 48 h post-transfection for collection and luciferase measurement was performed using Promega Dual-Glo (Promega #E2920), and a GloMax 96 microplate luminometer.

Pit1-zero and Pit1-triple cells were developed by Dr. Stephen Liebhaber and grown as described [31].

### Cloning

The DNA prepared for the plasmids used in the transfection experiments and transgenic mice was amplified from TαT1 DNA. Primers were designed to work with Phusion Green Hot Start II High-Fidelity PCR Master Mix (catalog # F566S). Two methods were employed to clone plasmids containing the regulatory element, the respective promoter, and the YFP reporter. The first method involved adding 10–15 nucleotides onto the insert that overlapped with the pCDNA3-YFP Basic plasmid that had been cut with Kpn1 and Xho1. The amplicon and linearized plasmid backbone were combined using the NEB HiFi DNA Assembly Master Mix (catalog # E2621L) with 1:2 vector:insert ratios, and 60 min incubation time at 50 °C. The subsequent plasmid was transformed into DH5α cells. The second method was to insert the amplified construct into a Zero Blunt TOPO vector (Thermo Fisher #450245), select clones that are in the forward and reverse orientation, then cut the TOPO vector containing the insert with Kpn1 and Xho1. The resulting fragment is then ligated into a digested pCDNA3-YFP Basic plasmid that had been cut with Kpn1 and Xho1 using a standard T4 DNA Ligase protocol (NEB #M0202). Once the insert, plasmid, and breakpoint sequences were confirmed by Sanger sequencing, the plasmids were extracted from overnight 1 L cultures of DH5α cells using Qiagen Plasmid Maxi Kits (catalog #12163). Endotoxins were removed from the plasmids using the Endotoxin removal Solution (Sigma, E4274-25ML).

### RNA-seq

One million GHF-T1 and TαT1 cells were collected for each of the three replicates for each cell line. Once collected, the RNA was extracted using the RNAqueous™ Total RNA Isolation Kit (catalog #AM1912). The RNA was prepared by the University of Michigan Advanced Genomic Core for mRNA enrichment followed by 50-cycle, paired-end sequencing on the Illumina HiSeq-4000. The RNA was checked for quality using FastQC and mapped and analyzed using the VIPER Snakemake pipeline [81]. Briefly, VIPER aligns the files to the mm9 transcriptome using STAR, followed by differential expression analysis using DESeq2 and cell type clustering and expression quantification using QoRTs [82]. The quality of the alignment was also analyzed using QoRTs.

We measured the significance of the increase in expression of sodium, potassium, and calcium using a one-way ANOVA. This demonstrated the significance of the increase in expression of the sodium and potassium, but not the calcium channel genes.

### GO term and KEGG pathway enrichment

We performed GO term enrichment on the top 5% of most differentially expressed genes (by log-2 fold-change) in both lines [32, 33]. This represented 453 genes in GHFT1 cells, and 490 genes in TαT1 cells. We used the default settings on the web-based Gene Ontology Resource (geneontology.org), using the biological process and *Mus musculus* options. The resulting GO terms were plotted by their log-2 fold enrichment, and their *p* values.

We also performed a directional Kyoto Encyclopedia of Genes and Genomes (KEGG) pathway enrichment analysis on all of the genes using RNA-enrich [83]. We set the maximum number of genes per concept to 500 and the minimum number of genes per concept to 5, and otherwise used the default settings. The resulting KEGG pathways were plotted by their coefficients and *p* values.

### ATAC-Seq

The Assay for Transposase-Accessible Chromatin with high-throughput sequencing (ATAC-seq) was performed as previously described [45, 84]. Briefly, 50,000 nuclei were extracted from collected GHF-T1 and TαT1 cells. The cells were transposed with Illumina transposase (Illumina #FC-121-1030) for 30 min at 37 °C while shaking at 250 RPM. The resulting fragmented DNA was amplified using ¼ of the cycles required to reach saturation in the described qPCR QC. The final amplified DNA library was purified using the Qiagen PCR purification kit (catalog #28104) and sequenced on the Illumina HiSeq platform. The quality of the reads was checked using FastQC, aligned to the mm9 genome, and had its peaks called using the Parker lab’s Snakemake pipeline [85].

### CUT&RUN

CUT&RUN was performed under high-digitonin conditions as described with few exceptions, namely all steps with < 1 ml of liquid requiring mixing were done by 500 RPM shaking instead of inversion [41]. Briefly, 250,000 cells per sample, and one sample per cell line-antibody pair were collected, washed, and bound to Concavalin A beads (Bangs Laboratories, BP531). The cells attached to beads were incubated at 4 °C overnight with the respective antibodies: POU1F1, Batch#1603-F, RRID=AB_2722652, provided by Dr. Simon Rhodes, University of North Florida, Jacksonville, FL [86]; H3K27Me3 – Cell Signal #9733, Batch #14, RRID=AB_2616029; H3K27Ac – Abcam ab4729, Batch GR3216173-1, RRID = AB_2118291; H3K4Me1 – Abcam ab8895, Batch GR3235544-1, RRID=AB_306847; Rabbit IGG – R&D AB-105-C, RRID= AB_354266). The antibodies were washed, and no secondary antibody was used. The protein A/MNAse fusion protein was added, followed by Ca^2+^-induced digestion at 0 °C for 30 min. The fragmented chromatin was then collected and purified using Macherey-Nagel NucleoSpin Gel and cleanup columns (catalog #740609). Libraries of this DNA was prepared using the Kapa Biosystems library prep kit (catalog #KK8702) at a 100:1 adapter to sample ratio. The libraries were paired-end sequenced on a single lane of the Illumina HiSeq-4000 for 50 cycles.

The resulting data was checked for quality using FastQC, aligned to the mm9 genome using Bowtie2 using the flags recommended for CUT&RUN (--local --very-sensitive-local --no-unal --no-mixed --no-discordant --phred33 -I 10 -X 700), and peaks were called using MACS2. We used ENCODE standards for evaluating the quality of our data. One of the metrics is the fraction of reads in peaks, or FRiP, which should correlate with the number of called regions, and it is generally accepted that values should be greater than 1% [87]. The FRiP values for GHFT1 and TαT1 cells, respectively, are as follows (in %): H3K27Ac = 21, 15; H3K4Me1 = 76, 78; POU1F1 = 32, 23; H3K27Me3 = 4, 6.

### ChromHMM

ChromHMM was performed on both cell lines using H3K4Me1, H3K27Ac, ATAC-seq, and H3K27Me3 as input. The number of states was iteratively increased to find the number of states that resulted in the fewest number of states with the best log-likelihood. An eleven-state model was selected as a result. Association of each state with the various marks and genomic features can be seen in Supplementary Figure 5. Contiguous states of value two and three were stitched together as enhancers using bedtool’s mergeBed function with a -d 1 flag [88].

### Association enrichment test

Enrichment analysis of disease SNPs at stretch enhancers in GHFT1, TαT1, and heterologous cell lines was performed using GARFIELD and stretch enhancers published previously [53, 55]. To find the human sequences orthologous to GHFT1 and TαT1 stretch enhancers, we used a conversion file generated using bnMapper and an mm9 to hg19 chain file [89]. We selected associations that had association counts of at least 50, had a full complement of summary statistics, had more than three million tested SNPs, and were in the harmonized data format were chosen from the GWAS catalog [90]. The resulting heatmap of odds ratios and *p* values for each association, tissue-type pair is shown in Supplementary Figure 7.

### Transgenic mice

All mice were housed in a 12-h light–12-h dark cycle in ventilated cages with unlimited access to tap water and Purina 5020 chow. All procedures were conducted in accordance with the principles and procedures outlined in the National Institutes of Health Guidelines on the Care and Use of Experimental Animals and approved by our Institutional Animal Care and Use Committee.

Recombinant DNA was generated by amplifying genomic mouse DNA regions in Supplemental Table 4, and the previously described Phusion polymerase. The putative regulatory element was then combined with 438 base pairs of the TSHβ promoter (chr3:102,586,594-102,587,032) and a YFP reporter. These elements were combined using the DNA Hifi reaction into a pGEM-T Easy plasmid. The putative regulatory element, the promoter, the YFP, and the breakpoints were checked for accuracy with Sanger sequencing. Once the plasmid was confirmed, larger quantities of the plasmid were generated from overnight 1 L cultures of DH5α cells using Qiagen Plasmid Maxi Kits (catalog #12163). To reduce the effect of the plasmid backbone on the viability of injected eggs, the enhancer, promoter, and YFP were amplified from the plasmid. The resulting amplicon was gel purified and injected into fertilized eggs of mice on a C57BL/6 and SJL mixed background. The resulting mice were genotyped for the YFP allele according to the Jackson Laboratory recommended primers and conditions [91]. We dissected the pituitaries from mice that were positive for the YFP transgene (and four age-matched, negative, control littermates) at 3 weeks of age.

### Tissue preparation and immunohistochemistry

Mouse pituitaries were fixed in 4% formaldehyde in PBS overnight at 4 °C. The tissue was washed three times in PBS and put in 10% EDTA for 3 h. They were then dehydrated by putting them in 25%, then 50%, and then 70% ethanol for 1 h each. The pituitaries were embedded in paraffin with 4-h cycles using a Tissue Tek VIP Paraffin tissue processing machine (Miles Scientific). The embedded pituitary was cut into coronal, six-micron sections, and was analyzed by immunohistochemical markers as previously described [22, 92]. Anti-YFP antibody (1:100) was from Abcam ab6556, Batch GR3216572-1, RRID=AB_305564, and anti-*Tshb* (1:1000) was from the National Hormone and Peptide Program, Batch AFP967793.

Antibodies were detected using either the tyramide signal amplification (TSA) (33002 CFF488A Streptavidin HRP, Biotium, Fremont, CA) and streptavidin-conjugated Alexa-fluor 488 (1:200, S11223, Invitrogen). DAPI (1:200) was incubated on the slides for 5 min to stain nuclei. DABCO-containing permount was used to mount the slides, which were then imaged using a Leica DMRB fluorescent microscope. To quantify gene expression, a set of 8–12 slides with pituitary sections were analyzed from each founder mouse. Total TSH positive cells/set were quantified, as well as total YFP-positive, and total double-positive cells expressing TSH and YFP. A total of 270–872 thyrotropes were counted for two founders.

## Supplementary Information

**Additional file 1: Figure S1.** Loss of ASCL1 has minimal impact on thyrotrope number. **Figure S2.** Multi-omics tracks for loci with similar levels of expression and chromatin landscapes in both cell types. **Figure S3.** Multi-omics tracks for selected genes with higher levels of expression in GHF-T1 cells. **Figure S4.** Multi-omics tracks for selected loci with higher levels of expression in TαT1 cells. **Figure S5.** ChromHMM summary data. **Figure S6.** Motif density at POU1F1 binding sites in GHF-T1 and TαT1 cells. **Figure S7.** Heatmap of associations with each cell type. **Figure S8.** Functional enhancer testing of elements of open chromatin in and around *Trhr.* **Figure S9.** Thyrotrope-specific genes identified by single cell sequencing are elevated in TαT1 cells relative to GHF-T1.

**Additional file 2: Table S1.** Genes Associated with SV40 Immortalized Pituitary Cell Lines. **Table S2.** bHLH genes expressed in GHF-T1 and TαT1 cells. **Table S3.** Thyrotrope signature genes. **Table S4.** Genomic coordinates for promoter and enhancer elements tested in transfection (Mm9). **Table S5.** Factors with binding motifs in *Tshb* Element 4. **Table S6.** Expression of reported thyrotrope-enriched genes in GHF-T1 and TαT1 cells.

## Data Availability

All data generated or analyzed during this study are included in this published article, its supplementary information files and publicly available repositories. All raw sequencing data generated in this study have been submitted to the NCBI Sequence Read Archive (SRA, https://www.ncbi.nlm.nih.gov/sra) under accession number PRJNA643917, SUB7682741.
