# Supplementary material for: Multi-omic profiling of pituitary thyrotropic cells and progenitors
Source: BMC Biol. 2021 Apr 15;19:76. doi: 10.1186/s12915-021-01009-0 (PMC8051135; doi:10.1186/s12915-021-01009-0)
Supplement: Supplementary file 1 — Additional file 1: Figure S1. Loss of ASCL1 has minimal impact on thyrotrope number. Figure S2. Multi-omics tracks for loci with similar levels of expression and chromatin landscapes in both cell types. Figure S3. Multi-omics tracks for selected genes with higher levels of expression in GHF-T1 cells. Figure S4. Multi-omics tracks for selected loci with higher levels of expression in TαT1 cells. Figure S5. ChromHMM summary data. Figure S6. Motif density at POU1F1 binding sites in GHF-T1 and TαT1 cells. Figure S7. Heatmap of associations with each cell type. Figure S8. Functional enhancer testing of elements of open chromatin in and around Trhr. Figure S9. Thyrotrope-specific genes identified by single cell sequencing are elevated in TαT1 cells relative to GHF-T1. [file 12915_2021_1009_MOESM1_ESM.docx]

**
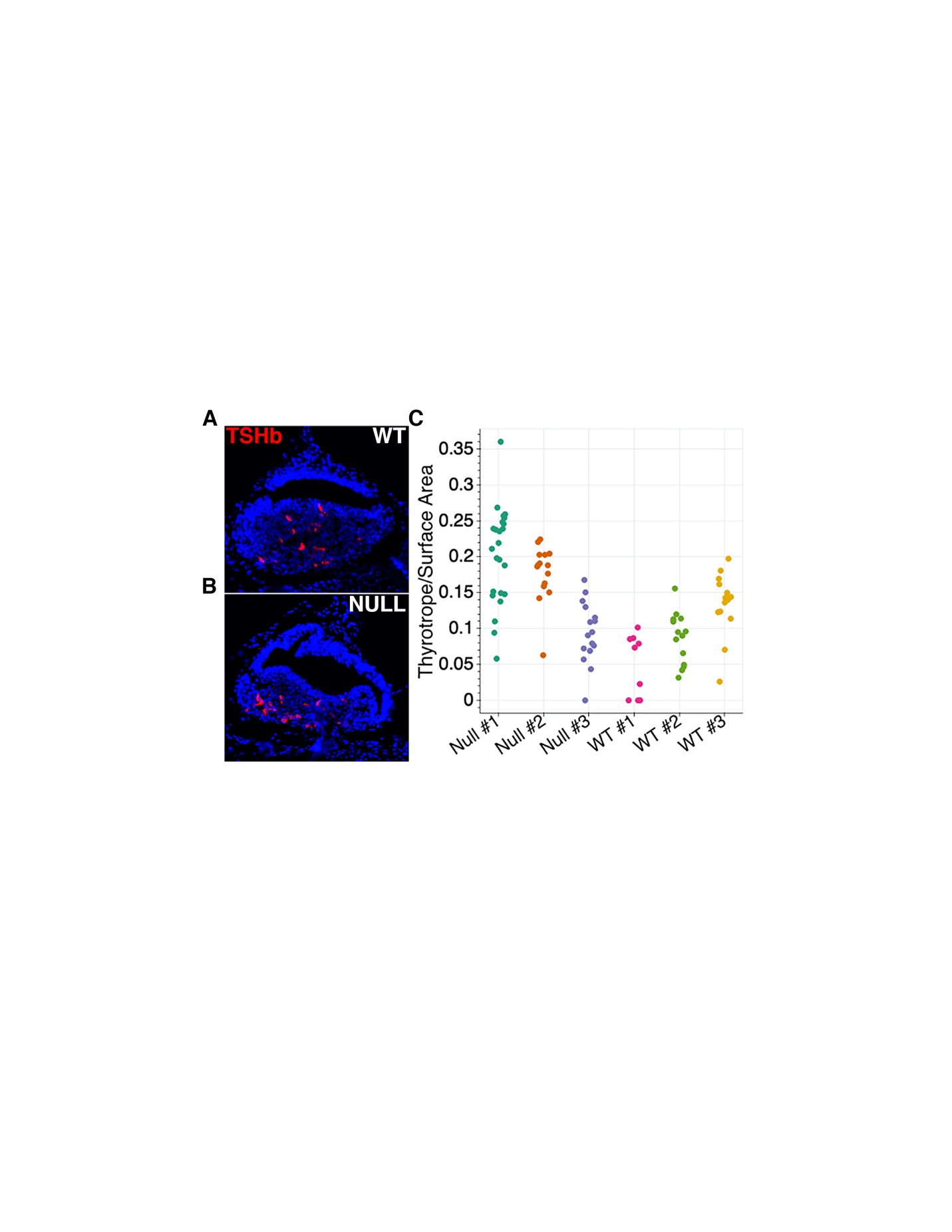
**

**Supplemental Figure 1: Loss of ASCL1 has minimal impact on thyrotrope number.**

(A) Immunostain for TSH (red) in sagittal section of a wild type e18.5 pituitary. (B) Immunostain for TSH (red) in *Ascl1*^-/-^ e18.5 pituitary. (C) Quantification of number of thyrotropes per surface area in individual sections throughout the pituitary gland in six e18.5 mice (three wild type, three null) revealing no significant difference between the genotypes (p-value = 0.71).

**
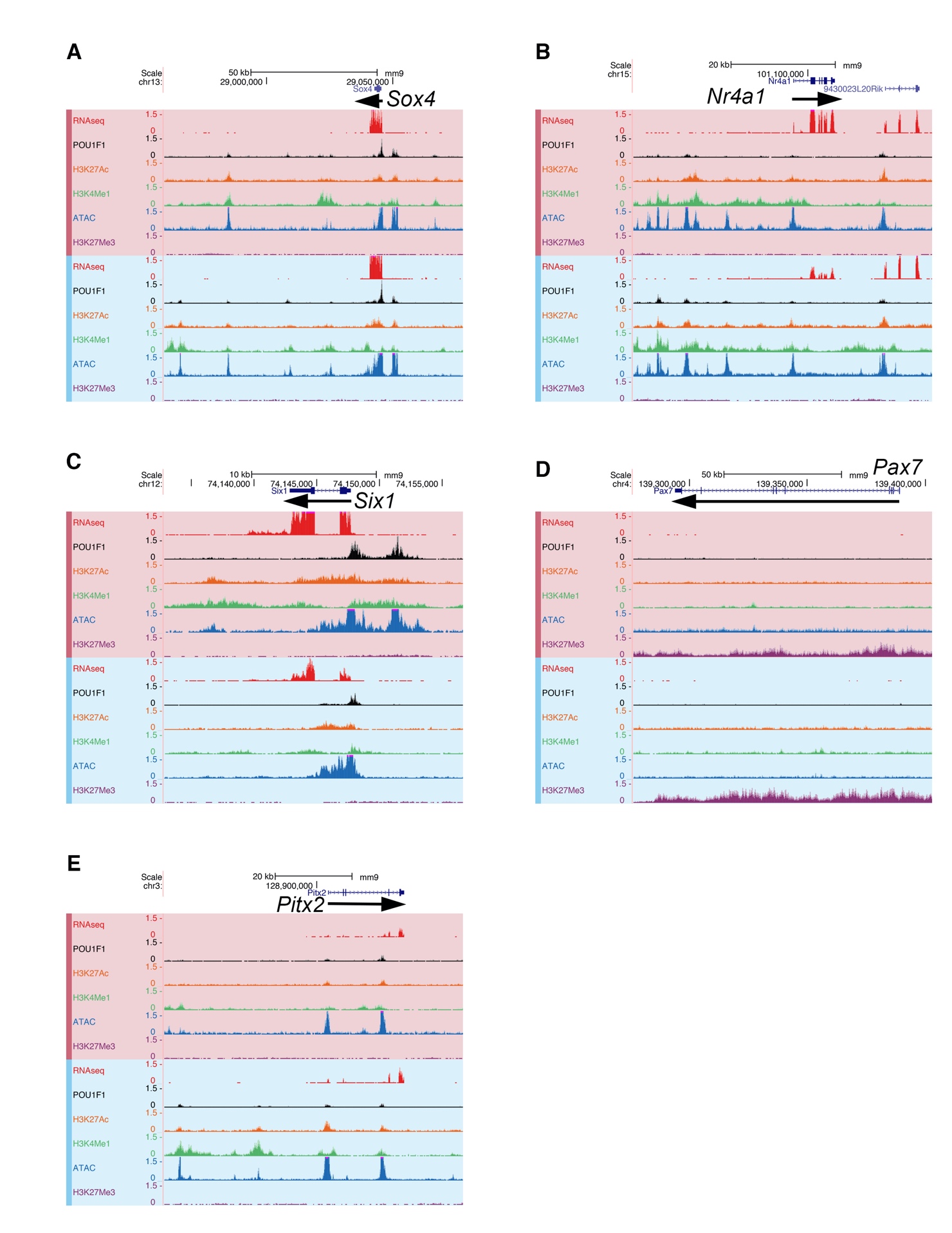
**

**Supplemental Figure 2: Multi-omics tracks for loci with similar levels of expression and chromatin landscapes in both cell types**.

RNA-seq, POU1F1, H3K27Ac, H3K4Me1, ATAC-seq, and H3K27Me3 tracks at (**A**) *Sox4*, (**B**) *Nr4a1*, (**C**) *Six1*, (**D**) *Pax7*, and (**E**) *Pitx2* loci in TαT1 cells (red) and GHF-T1 (blue).

**
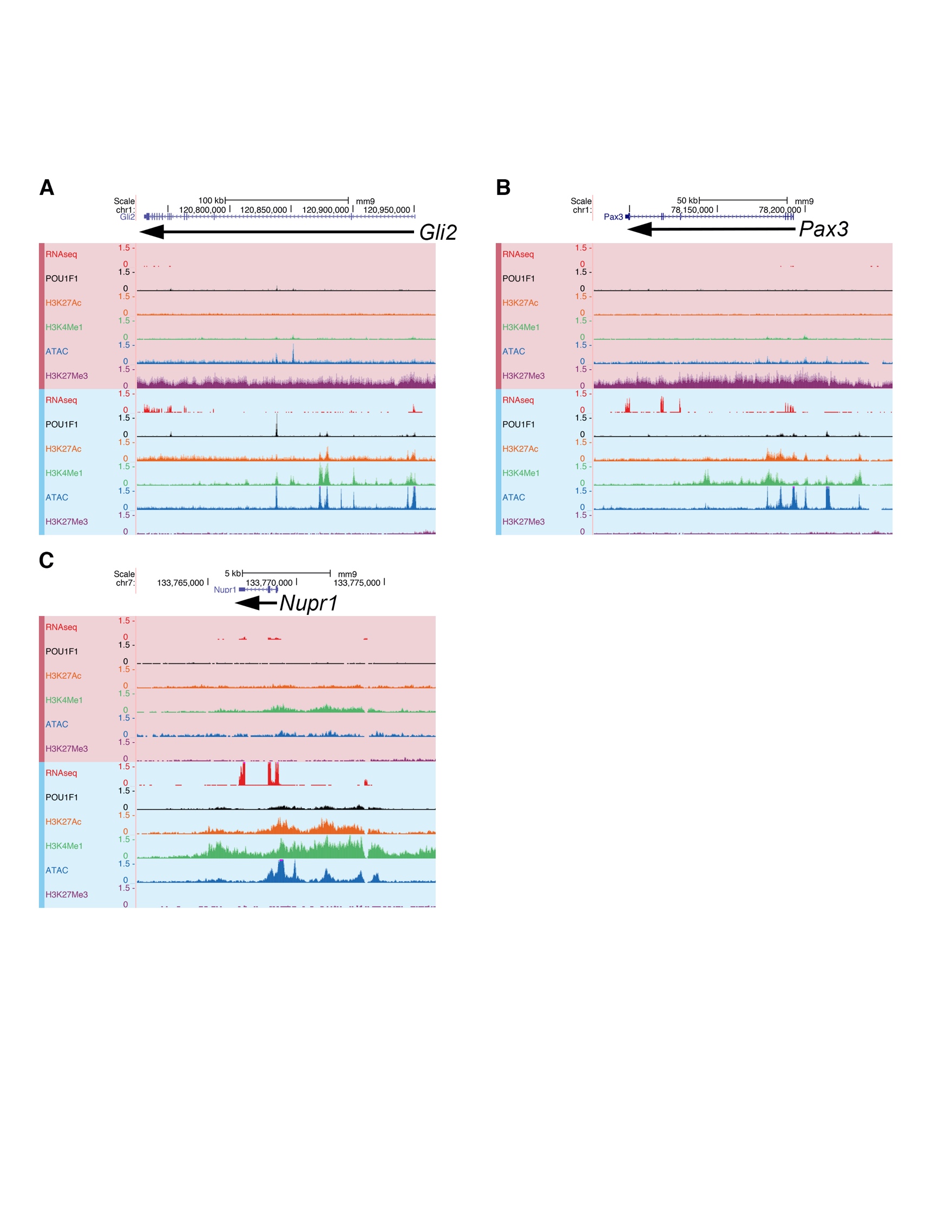
**

**Supplemental Figure 3: Multi-omics tracks for selected genes with higher levels of expression in GHF-T1 cells than TαT1.**

RNA-seq, POU1F1, H3K27Ac, H3K4Me1, ATAC-seq, and H3K27Me3 tracks in TαT1 (red) and GHF-T1 (blue) cells for (**A**) *Gli2*, (**B**) *Pax3*, and (**C**) *Nupr1*.

**
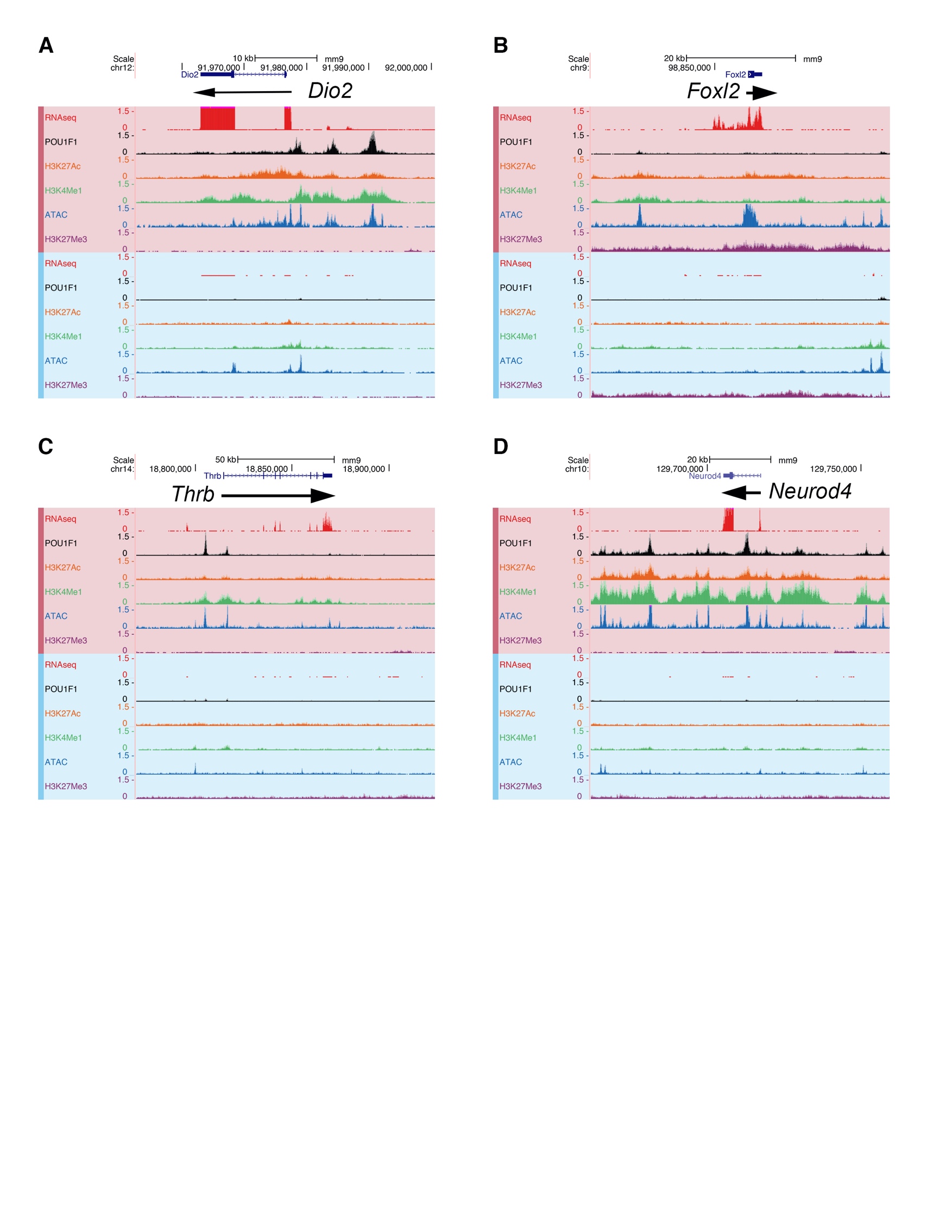
**

**Supplemental Figure 4: Multi-omics tracks for selected loci with higher levels of expression in TαT1 cells than GHFT1**.

RNA-seq, POU1F1, H3K27Ac, H3K4Me1, ATAC-seq, and H3K27Me3 tracks from TαT1 (red) and GHF-T1 (blue) cells at (**A**) *Dio2*, (**B**) *Foxl2*, (**C**) *Thrb*, and (**D**) *Neurod4*.

**
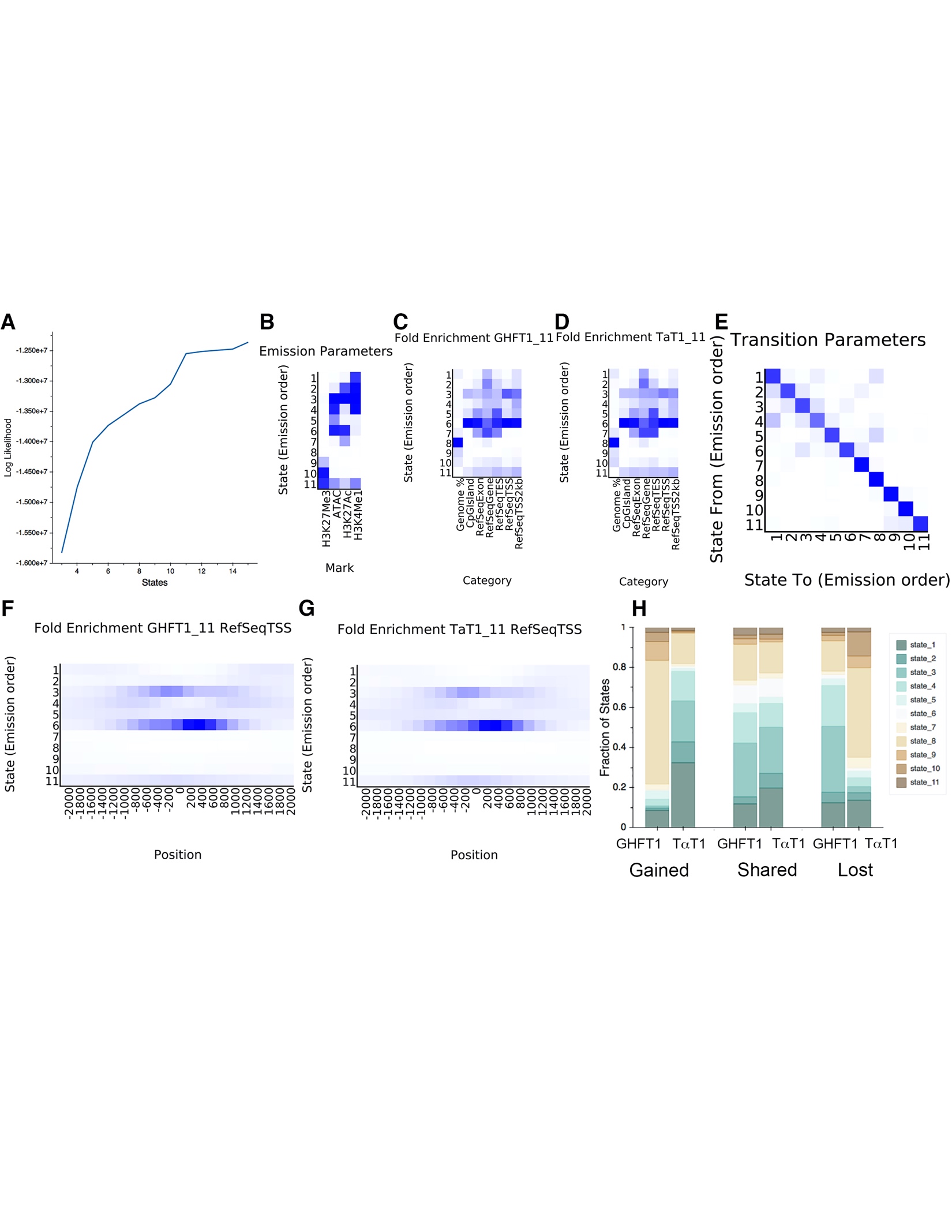
**

**Supplemental Figure 5: ChromHMM summary data**.

(**A**) Log likelihood of the resulting ChromHMM performed for each number of states. Eleven states had the best combination of the fewest states and most favorable log likelihood. (**B**) The emission parameters reveal the presence of each histone mark and the level of ATAC-seq signal for each state with darkest blue being the highest enrichment. (**C**) Genomic features of each state in GHF-T1 cells. (**D**) Genomic features of each state in TαT1 cells. (**E**) A plot revealing the degree of transition from each parameter to each other parameter. (**F**) Degree of enrichment of each state at TSS’s in GHF-T1 cells, revealing that state six is heavily enriched at promoters. (**G**) Degree of enrichment of each state at TSS’s in TαT1 cells, revealing that state six is heavily enriched at promoters. (**H**) In-depth breakout of Figure 4G. Composition of TαT1-specific POU1F1 binding site chromatin states in GHF-T1 and TαT1 cells (gained sites, left), composition of shared POU1F1 binding site chromatin states in GHF-T1 and TαT1 cells (shared sites, center), composition of GHF-T1-specific POU1F1 binding site chromatin states in GHF-T1 and TαT1 cells (lost sites, right).

**
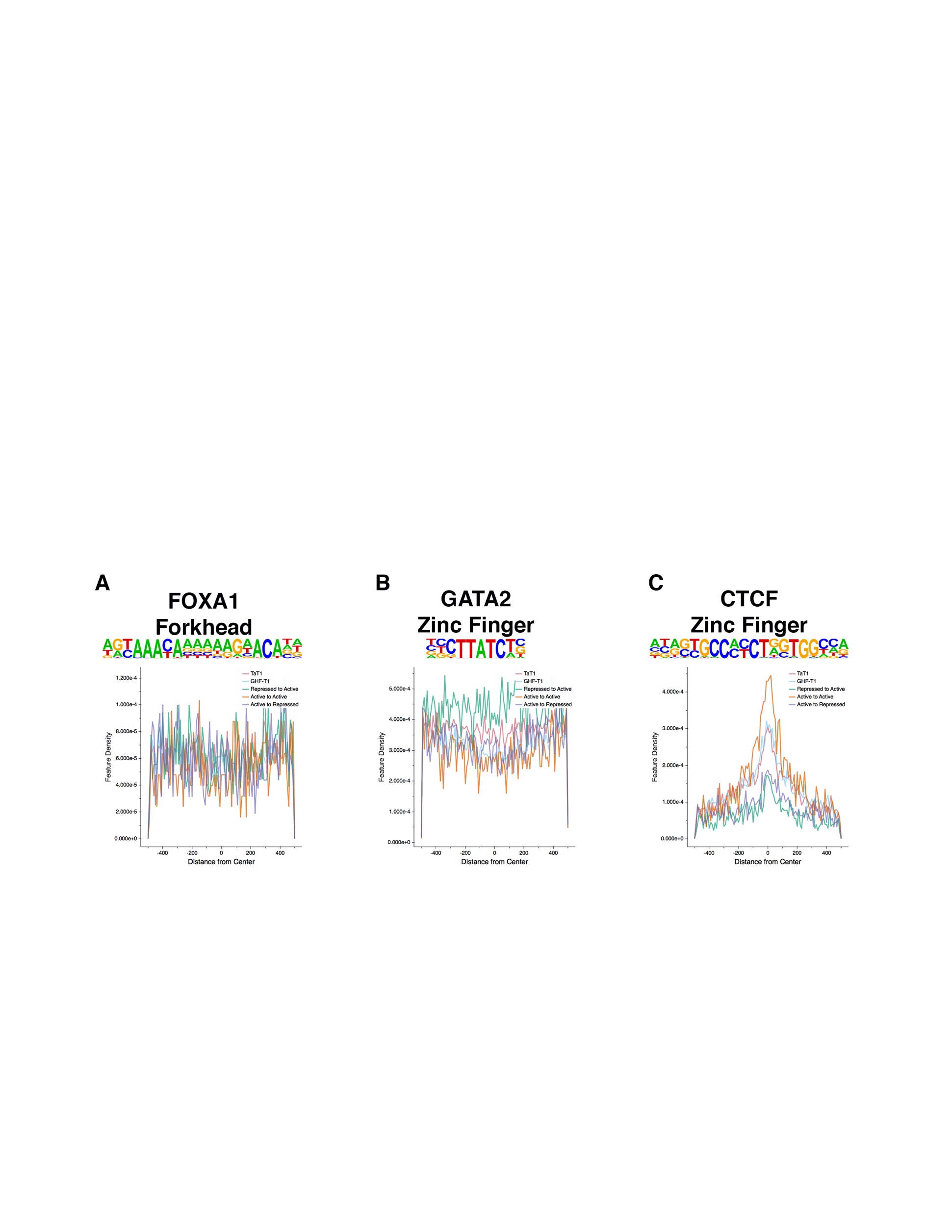
**

**Supplemental Figure 6: Motif density for selected transcription factors at POU1F1 binding sites in GHF-T1 and TαT1 cells**.

(A) Motif density of the representative Forkhead factor FOXA1 at all TαT1 POU1F1 binding sites (red), at all GHF-T1 POU1F1 binding sites (blue), at POU1F1 binding sites that are specific to TαT1 that have repressive marks in the GHF-T1 precursor cells and active chromatin in the differentiated, TαT1 cells (Repressed to Active, green), at POU1F1 binding sites that are shared between both lines and have similarly active chromatin marks in both (Active to Active, orange), and POU1F1 binding sites that are specific to GHF-T1 that have active chromatin in GHF-T1 cells and repressed chromatin in TαT1 cells (Active to Repressed, purple). (B) Motif density of GATA2 at the same POU1F1 binding domains as in (A). (C) Motif density of CTCF at the same POU1F1 binding domains as in (A).

**
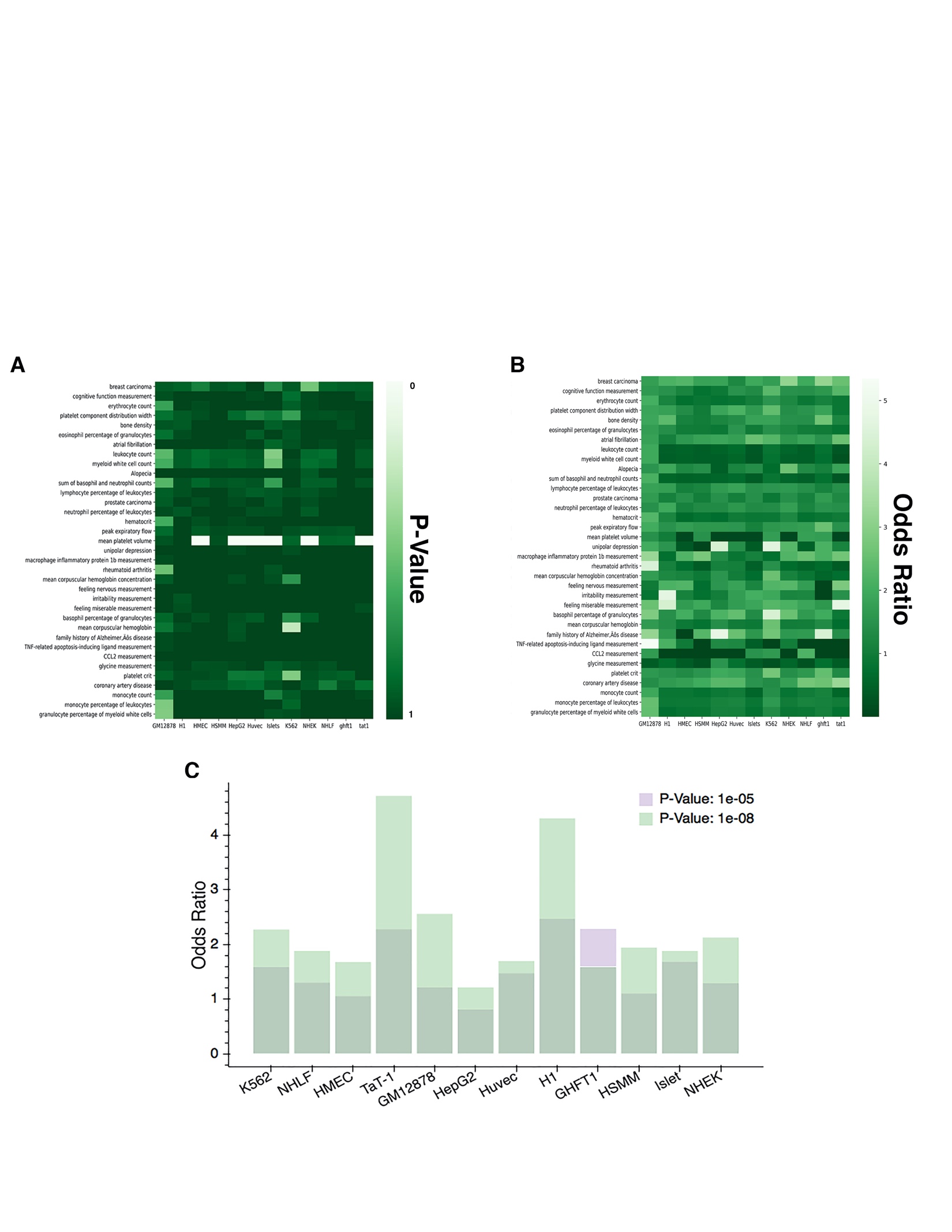
**

**Supplemental Figure 7: Heatmap of associations with each cell type**.

(A) P-value for each enrichment test performed for each association and cell type pair. (B) Odds ratio for each enrichment test performed for each association and cell type pair. (C) The odds ratio of observing such an enrichment of SNPs for the neuroticism sub-phenotype of feeling miserable within stretch enhancers of all tissues tested.

**
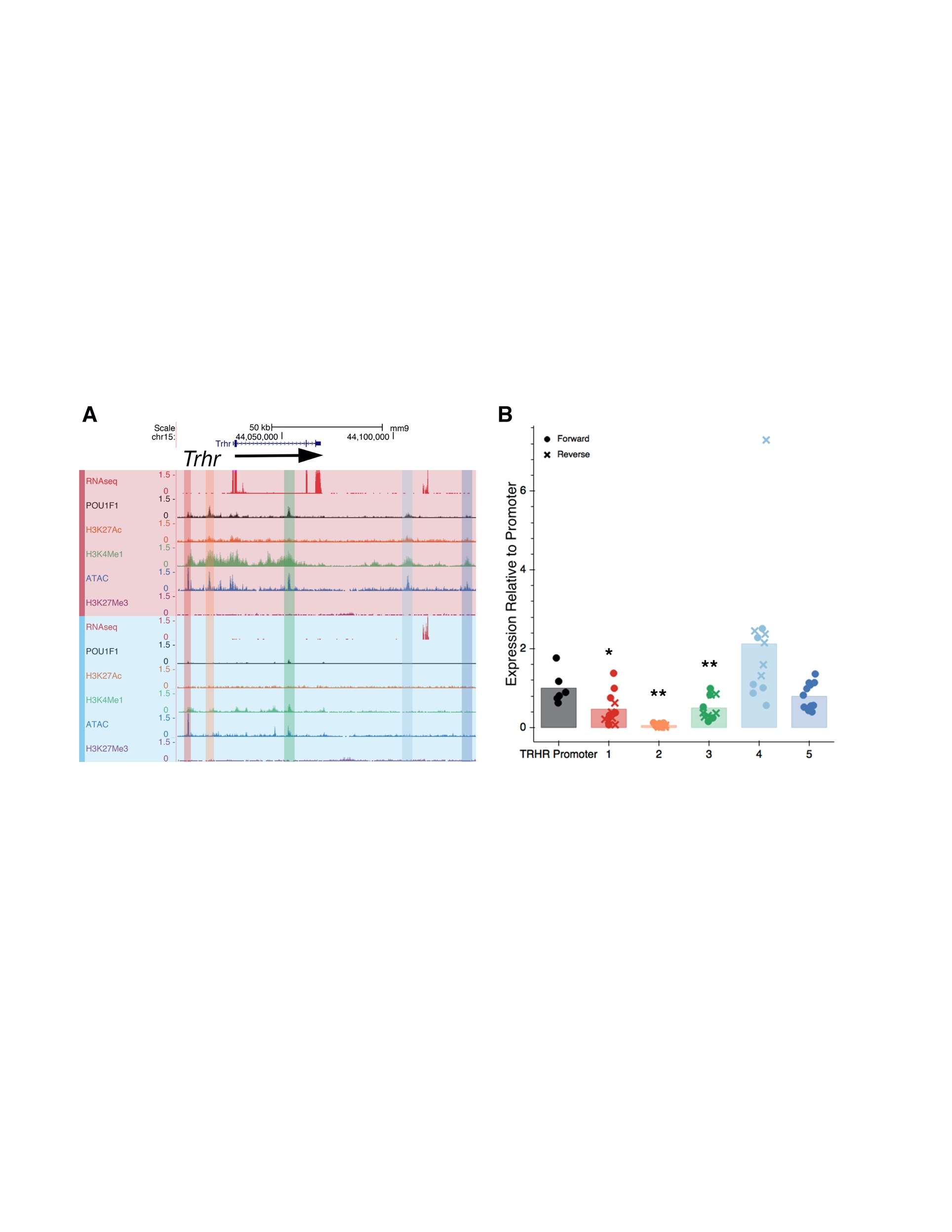
**

**Supplemental Figure 8: Functional enhancer testing of elements of open chromatin in and around *Trhr***

(A) RNA-seq, POU1F1, H3K27Ac, H3K4Me1, ATAC-seq, and H3K27Me3 tracks (TαT1 in red, GHF-T1 in blue) at the *Trhr* locus where elements tested are highlighted. (B) Level of luciferase activity of each element, color-coordinated with the highlighted elements in **A** in both the forward (circles) and reverse (x’s) orientation. (p-value < 0.05 = *; p-value < 0.01 = **)

**
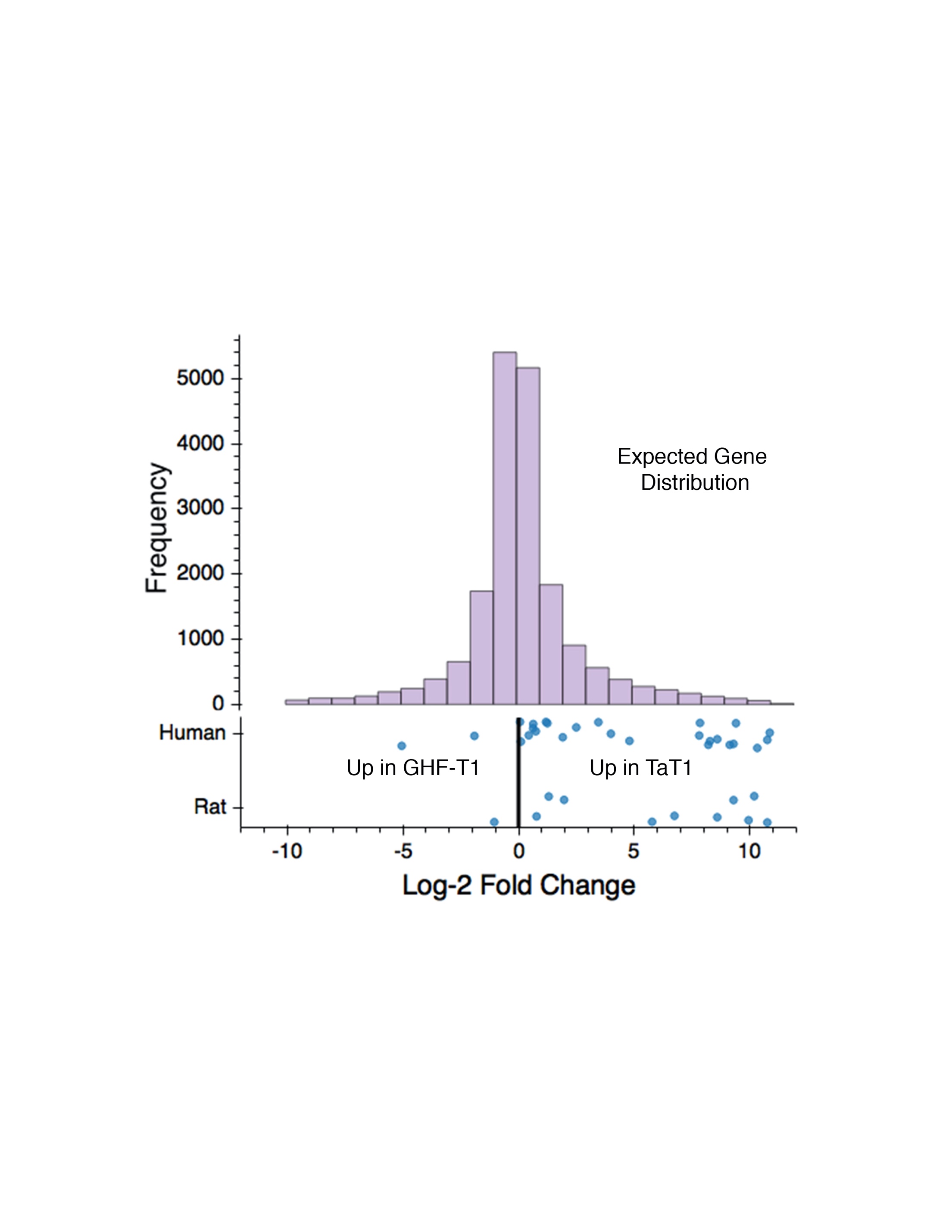
**

**Supplemental Figure 9: Thyrotrope-specific genes identified by single cell sequencing are elevated in TαT1 cells relative to GHF-T1.**

Thyrotrope-enriched genes identified in single cell RNA sequencing data from pituitaries of adult rats and human fetuses were evaluated for expression levels in GHF-T1 and TαT-1 cells and plotted as log-2-fold change. The frequency histogram shows the expectation for a random distribution across the spectrum of differential expression. Thus, the majority of thyrotrope-enriched genes exhibited elevated expression in TαT-1 cells. Only *Sox4*, identified in human embryonic thyrotropes, and *Arg1* and *Prodh*, identified in rat thyrotropes, exhibited elevated expression in GHF-T1 relative to TαT-1.
