# Supplementary material for: Multi-omic profiling of pituitary thyrotropic cells and progenitors
Source: BMC Biol. 2021 Apr 15;19:76. doi: 10.1186/s12915-021-01009-0 (PMC8051135; doi:10.1186/s12915-021-01009-0)
Supplement: Supplementary file 2 — Additional file 2: Table S1. Genes Associated with SV40 Immortalized Pituitary Cell Lines. Table S2. bHLH genes expressed in GHF-T1 and TαT1 cells. Table S3. Thyrotrope signature genes. Table S4. Genomic coordinates for promoter and enhancer elements tested in transfection (Mm9). Table S5. Factors with binding motifs in Tshb Element 4. Table S6. Expression of reported thyrotrope-enriched genes in GHF-T1 and TαT1 cells. [file 12915_2021_1009_MOESM2_ESM.docx]

Supplemental Tables

Supplemental Table 1: Genes Associated with SV40 Immortalized Pituitary Cell Lines.

| **Cluster 1**  **(Pit1-Zero + Pit1-Triple** | **Cluster 2 (GHF-T1)** | **Cluster 3 (TαT1)** |
| --- | --- | --- |
| *Lxn* | *Fabp7* | *Cga* |
| *Tbx18* | *Sdpr* | *Nnat* |
| *Mir692-1* | *Vax1* | *Pcp4* |
| *Gypc* | *Shtn1* | *Smtnl2* |
| *Arsj* | *Bst2* | *Chga* |
| *Pbp2* | *Tuba8* | *Chgb* |
| *Lce1g* | *Gas7* | *Pnmal1* |
| *Ereg* | *Tprg* | *Resp18* |
| *Lox* | *Cox7a1* | *Elavl3* |
| *Nid1* | *Ldhb* | *Pcsk2* |
| *Ces1g* | *Lrrc17* | *Syp* |
| *Grem1* | *1700019N19Rik* | *Scg3* |
| *Bgn* | *Chrnb1* | *Scg2* |
| *Gja1* | *Cgnl1* | *Sez6l2* |
| *Chrna1* | *Clvs2* | *Celf3* |
| *Casp8* | *Btbd3* | *Snap25* |
| *Hebp2* | *Rcn3* | *Cdk5r2* |
| *Pdgfrb* | *Hotairm1* | *Zim1* |
| *Ptx3* | *Creb5* | *Scg5* |
| *Tpm2* | *Meis2* | *Insm1* |

Supplemental Table 2: bHLH genes expressed in GHF-T1 and TαT1 cells.

| **Gene** | **GHF-T1 Expression (FPKM)** | **TαT1 Expression (FPKM)** |
| --- | --- | --- |
| **Shared Expression** | | |
| *Id2* | 155.4 | 253.8 |
| *Id1* | 121.2 | 203.8 |
| *Tcf4* | 99.6 | 166.6 |
| *Hif1a* | 49.92 | 103.4 |
| *Tcf12* | 48.4 | 83.2 |
| *Bhlhb9* | 33.1 | 82.6 |
| *Srebf2* | 61.1 | 44.7 |
| *Tcf3* | 50.2 | 41.9 |
| *Bhlhe40* | 23.3 | 69 |
| *Max* | 45.4 | 41.1 |
| **Elevated in GHF-T1** | | |
| *Tcf24* | 2.3 | 0 |
| *Twist2* | 6.6 | 0 |
| *Bhlhe22* | 4.8 | 0.1 |
| *Twist1* | 17.3 | 0.5 |
| *Atoh8* | 42.2 | 2.9 |
| *Msc* | 3.2 | 0.3 |
| *Hey2* | 2.3 | 0.3 |
| *Hes1* | 16.6 | 2.6 |
| *Epas1* | 3.3 | 0.7 |
| *Id3* | 226.7 | 63.5 |
| **Elevated in TαT1** | | |
| *Neurod4* | 0 | 31.9 |
| *Ascl1* | 0.2 | 25.3 |
| *Olig1* | 0.1 | 3.7 |
| *Mycn* | 0.3 | 13.9 |
| *Heyl* | 0 | 1.3 |
| *Id4* | 3.3 | 81.3 |
| *Tal1* | 0.1 | 2.7 |
| *Mlxipl* | 0.3 | 2.6 |
| *Ncoa1* | 2.1 | 13.5 |
| *Arntl* | 10.4 | 41.5 |

Supplemental Table 3: Thyrotrope signature genes. Expression values are in FPKM.

| **Gene** | **Protein** | **Expression values** (FPKM) | | **References**  (PMIDs) |
| --- | --- | --- | --- | --- |
|  |  | **GHF-T1** | **TαT1** |  |
| *Cga* | Chorionic gonadotropin alpha subunit | 1.46 | 3556.96 | 7544315 |
| *Creb3l1* | cAMP-responsive element-binding protein 3-like 1 | 5.89 | 172.46 | 29311806, 27580811 |
| *Dio2* | Deiodinase 2 | 0.19 | 125.99 | 11731615, 5007895 |
| *E2f1* | E2F transcription factor 1 | 35.70 | 50.09 | 27580811, 18794899 |
| *Etv5* | Ets variant 5 | 13.30 | 20.00 | 27580811, 19898483, 16107850 |
| *Eya3* | Eyes absent transcriptional co-activator and phosphatase 3 | 15.87 | 11.53 | 21129973 |
| *Foxl2* | Forkhead transcription factor FOXL2 | 0.07 | 18.14 | 11175783, 12149404, 15056605, 14736745, 29800110, 16840539 |
| *Gata3* | GATA binding protein 3 | 0.80 | 0.49 | 16543408, 10935639 |
| *Isl1* | Islet 1 | 17.60 | 45.67 | 32453714, 27580811 |
| *Lhx2* | LIM homeodomain transcription factor 2 | 2.60 | 35.80 | 7513049, 19900438 |
| *Lhx3* | LIM homeodomain protein 3 | 0.01 | 44.22 | 8638120, 16394081 |
| *Msx1* | Muscle segment homeodomain 1 | 5.23 | 15.95 | 12807959, 7914451, 23371388, 1837990/0,  16703404 |
| *Nr4a1* | Nuclear receptor subfamily 4, group a, member 1 (Nurr77) | 9.29 | 33.65 | 22792320, 30093910 |
| *Nupr1* | Nuclear protein transcriptional regulator 1 | 41.97 | 1.93 | 12429736, 18495683, 27580811 |
| *Pitx1* | Paired homeodomain transcription factor 1 | 109.26 | 363.52 | 21775501, 10049363,10101115, 15761027 |
| *Pitx2* | Paired homeodomain transcription factor 2 | 6.97 | 4.28 | 8944018, 10498698, 11807026 |
| *Pou1f1* | POU homeodomain transcription factor (PIT1) | 85.11 | 159.63 | 1302000, 15928241, 1981057,1977085 |
| *Rxrg* | Retinoid receptor X gamma | 0.01 | 25.55 | 16306084, 108800050 |
| *Six1* | Sine oculis related homeodomain transcription factor 1 | 11.55 | 45.30 | 9020840, 1978983, 14628042 |
| *Sox4* | SRY-box 4 | 36.14 | 18.35 | 22543271, 30661772, 9815146 |
| *Tceal5* | Transcription elongation factor A (SII)-like 5 | 0.04 | 0.01 | 27580811 |
| *Tef* | Thyrotroph embryonic factor | 12.78 | 24.65 | 1916262, 15175240 |
| *Thrb* | Thyroid hormone receptor beta | 0.16 | 9.07 | 22570333, 32122258 |
| *Trhr* | Thyrotropin releasing hormone receptor | 0.02 | 39.54 | 9141550, 14988432 |
| *Tshb* | Thyroid stimulating hormone beta subunit | 0.01 | 11.06 | 2792087 |

Supplemental Table 4:
Genomic coordinates for promoter and enhancer elements tested in transfection (Mm9).

| **Gene** | **Chromosome** | **Element** | **Start** | **Stop** |
| --- | --- | --- | --- | --- |
| *Gata2* | 6 | 200 bp Promoter | 88148535 | 88148762 |
|  |  | 900 bp Promoter | 88147851 | 88148762 |
|  |  | 2.8 kb Promoter | 88145925 | 88148762 |
|  |  | Element 1 (2.8 kb) | 88139279 | 88141082 |
|  |  | Element 2 (1.8 kb) | 88176353 | 88177232 |
|  |  | Element 3 (1.2 kb) | 88261331 | 88262559 |
| *Cga* | 4 | Promoter (486 bp) | 34840577 | 34841063 |
|  |  | Element 1 (1.8 kb) | 34833573 | 34835422 |
|  |  | Element 2 (964 bp) | 34836822 | 34837786 |
|  |  | Element 3 (1.3 kb) | 34846587 | 34847868 |
| *Pitx1* | 13 | Promoter (377 bp) | 55932587 | 55932964 |
|  |  | Element 1 (3.1 kb) | 55941401 | 55944503 |
|  |  | Element 2 (1.9 kb) | 55951439 | 55953329 |
| *Tshb* | 3 | Promoter (438 bp) | 102586594 | 102587032 |
|  |  | Element 1 (1.9 kb) | 102527463 | 102529377 |
|  |  | Element 2 (3.9 kb) | 102536553 | 102540450 |
|  |  | Element 3 (3.3 kb) | 102550639 | 102553942 |
|  |  | Element 4 (1.4 kb) | 102592924 | 102594331 |
|  |  | Element 5 (4.4 kb) | 102605012 | 102609378 |
| *Trhr* | 15 | Promoter (957 bp) | 44027215 | 44028172 |
|  |  | Element 1 (4.3 kb) | 44007238 | 44011587 |
|  |  | Element 2 (4.9 kb) | 44016635 | 44021495 |
|  |  | Element 3 (4.4 kb) | 44050106 | 44054542 |
|  |  | Element 4 (3.8 kb) | 44105357 | 44109171 |
|  |  | Element 5 (1.2 kb) | 44132604 | 44133764 |

Supplemental Table 5: Factors with binding motifs in *Tshb* Element 4.

| **Gene** | **Motif** | | | **TαT1 Expression** |
| --- | --- | --- | --- | --- |
|  | **Number** | **Top Score** | **Average Score** |  |
| **Sorted by TαT1 Expression** | | | | |
| *Pitx1* | 31 | 8 | 5 | 363.5 |
| *Maz* | 2 | 16 | 11 | 187.3 |
| *Tcf4* | 21 | 13 | 5 | 166.6 |
| *Pou1f1* | 5 | 10 | 10 | 159.6 |
| *Atf4* | 7 | 10 | 8 | 147.2 |
| *Cenpb* | 2 | 5 | 5 | 114.4 |
| *Hif1a* | 4 | 6 | 5 | 103.4 |
| *Tfdp1* | 1 | 9 | 9 | 97.6 |
| *Tcf12* | 17 | 15 | 5 | 83.2 |
| *Plagl1* | 1 | 7 | 7 | 78.6 |
| *Jund* | 19 | 8 | 3 | 77.5 |
| *Bhlhe40* | 6 | 8 | 4 | 69 |
| *Mef2a* | 27 | 11 | 7 | 66.4 |
| *Smad4* | 6 | 5 | 5 | 58.2 |
| *Stat5a* | 7 | 11 | 10 | 57.1 |
| *Pknox2* | 2 | 11 | 11 | 53.5 |
| *Ctcf* | 1 | 9 | 9 | 51.4 |
| *E2f1* | 3 | 6 | 5 | 50.1 |
| *Vezf1* | 4 | 7 | 6 | 47 |
| *Isl1* | 15 | 11 | 6 | 45.7 |
| **Ranked by Number of motifs (TαT1 FPKM >=1)** | | | | |
| *Foxd2* | 76 | 7 | 4 | 2 |
| *Lhx9* | 76 | 11 | 5 | 1.3 |
| *Hoxa5* | 75 | 11 | 5 | 1.3 |
| *Gata2* | 65 | 14 | 5 | 34.8 |
| *Foxc1* | 60 | 9 | 4 | 4.8 |
| *Lhx4* | 60 | 10 | 5 | 1.1 |
| *Lhx1* | 58 | 9 | 5 | 8.5 |
| *Arid3a* | 51 | 9 | 5 | 1.2 |
| *Stat3* | 50 | 11 | 4 | 30 |
| *Lin54* | 49 | 13 | 6 | 22.6 |
| *Hltf* | 49 | 8 | 5 | 6.1 |
| *Dlx1* | 48 | 9 | 5 | 2.1 |
| *Hlf* | 47 | 12 | 7 | 36.7 |
| *Ets1* | 47 | 12 | 5 | 3.1 |
| *Nkx3-2* | 47 | 12 | 6 | 1.7 |
| *Tbp* | 46 | 10 | 7 | 24.9 |
| *Vax2* | 43 | 11 | 5 | 10.6 |
| *Zeb1* | 43 | 11 | 5 | 9.4 |
| *Klf4* | 43 | 10 | 6 | 7.7 |
| *Hoxa6* | 42 | 9 | 5 | 1.3 |

| **Supplemental Table 6. Expression of reported thyrotrope-enriched genes in GHF-T1 and TαT1 cells** | | | |
| --- | --- | --- | --- |
| **Sample, gene** | **GHF-T1**  (FPKM) | **TαT1**  (FPKM) | **Log 2-fold change** |
| **Human fetus** |  |  |  |
| *Ascl1* | 0.22 | 25.32 | 6.73 |
| *Dio2* | 0.19 | 125.99 | 9.29 |
| *Fev* | 0.00 | 10.26 | 10.18 |
| *Gata2* | 8.46 | 34.78 | 1.97 |
| *Isl1* | 17.60 | 45.67 | 1.30 |
| *Myt1l* | 0.01 | 14.64 | 9.93 |
| *Rxrg* | 0.01 | 25.55 | 10.74 |
| *Sox11* | 10.82 | 19.47 | 0.78 |
| *Sox4* | 36.14 | 18.35 | -1.05 |
| *Thrb* | 0.16 | 9.07 | 5.77 |
| *Tshb* | 0.01 | 11.06 | 8.58 |
| **Adult rat** | | | |
| *Adam23* | 1.55 | 6.11 | 1.91 |
| *Arg1* | 0.82 | 0.02 | -5.04 |
| *Atp6ap1l* | 0.00 | 0.03 | 1.18 |
| *Bdnf* | 1.61 | 2.30 | 0.44 |
| *Bmp15* | 0.00 | 0.00 | na |
| *Dio2* | 0.19 | 125.99 | 9.29 |
| *Dnah11* | 0.00 | 0.02 | 2.49 |
| *Dpp10* | 0.00 | 3.15 | 9.12 |
| *Fam183b* | 0.00 | 14.57 | 9.39 |
| *Gdf11* | 14.22 | 23.19 | 0.63 |
| *Gpr158* | 0.01 | 3.30 | 7.84 |
| *Grem1* | 0.04 | 0.07 | 0.63 |
| *Irs4* | 0.32 | 5.38 | 3.99 |
| *Nell1* | 0.00 | 6.00 | 10.31 |
| *Nmu* | 0.02 | 0.09 | 1.25 |
| *Nrg4* | 0.08 | 1.02 | 3.45 |
| *Parm1* | 0.71 | 20.78 | 4.79 |
| *Pcsk1* | 0.03 | 12.06 | 8.2 |
| *Pcsk2* | 0.23 | 75.22 | 8.27 |
| *Pgm2l1* | 6.73 | 11.85 | 0.75 |
| *Prodh* | 0.38 | 0.10 | -1.91 |
| *Rxrg* | 0.01 | 25.55 | 10.74 |
| *Trhr* | 0.02 | 39.54 | 10.85 |
| *Trnp1* | 10.29 | 11.60 | 0.1 |
| *Tshb* | 0.01 | 11.06 | 8.58 |
| *Zcchc16* | 0.00 | 0.65 | 7.8 |
| *Zfp365* | 5.91 | 6.49 | 0.06 |
